# Analysis of the shape characteristics and nutritional components of *Akebia trifoliata* in Qinba Mountains

**DOI:** 10.3389/fpls.2022.975677

**Published:** 2022-09-29

**Authors:** Min Wang, Xiaocheng Guo, Junyang Song

**Affiliations:** ^1^ College of Landscape Architecture and Art, Northwest Agriculture and Forestry (A&F) University, Xianyang, China; ^2^ Xi’an Agricultural Technology Promotion Center, Xi'an, China

**Keywords:** *Akebia trifoliata*, fruit, appearance characteristics, nutritional components, new varieties

## Abstract

*Akebia trifoliata (A. trifoliata)* is a widely distributed wild vine that has attracted much attention in recent years due to the edible fruit of food and medicinal value. In this paper, the fruits of *A. trifoliata*, which are derived from Qinling Mountains (12 genotypes) and Bashan Mountains (4 genotypes) and have been artificially domesticated and cultivated for many years, are taken as the research object to study the fruit characteristics and pulp components of 16 genotypes of *A. trifoliata*. The results show that the pulp of the 16 genotypes contain a variety of nutrients, among which the average contents of total sugar, total acid, vitamin C, soluble solids and starch are 14.68g/100g, 0.14g/100g, 26.40mg/100g, 17.95% and 5.29g/100g. The fruit contains 17 amino acids, including 7 essential amino acids and 4 organic acids. The latter refers to malic acid, lactic acid, citric acid and fumaric acid, the average contents of which are 1.03g/kg, 3.38g/kg, 0.33g/kg and 0.0149g/kg. Besides, 8 mineral elements in the fruit include 4 macro elements and 4 micro elements. The average contents of the former are potassium (1.83g/kg), calcium (0.23g/kg), phosphorus (0.28g/kg) and magnesium (0.21g/kg), and the average contents of the latter are iron (2.29mg/kg), zinc (2.23mg/kg), copper (1.37mg/kg) and manganese (5.52mg/kg). During the ripening process of *A. trifoliata* fruit (using HY-9 as the material), the main nutrients in the pulp such as total sugar, soluble solids, starch, amino acids and various mineral elements reach the maximum in stage 3, indicating that stage 3 is the best edible period of *A. trifoliata* fruit. Through the assignment analysis and comprehensive evaluation of 9 quality indicators (3 apparent characters and 6 main chemical components) of the fruits of the 16 A*. trifoliata* genotypes from Qinba Mountains, HY-1, HY-2 and HY-9 were finally screened out as the three superior genotypes. This study aims to provide reference for the development and utilization of *A. trifoliata* wild germplasm resources and the selection of new varieties.

## 1 Introduction

*Akebia trifoliata* [*Akebia trifoliata* (Thunb.) Koidz], a wild woody intertwining vine of the genus *Akebia* in the family Lardizabalaceae (Li et al., 2018; Zou et al., 2019), often grows on the edge of forests, sparse forests along mountains and valleys, dense forests or sparse forests and shrubs with relatively shady wetlands (Zou et al., 2019), and bears fruits which are commonly known in China as August Melon, August Fried, Wild Banana, etc. (Zhang et al., 2016; Niu et al., 2019). It is also a famous medicinal plant in China with moderate properties and slightly bitter taste (Xie et al., 2006; Li et al., 2009). The seeds, fruits, vines and roots have been used as traditional Chinese medicine for more than 2,000 years (Li et al., 2006; Feng, 2010; Jiang et al., 2020; Yu et al., 2020) and were included in the *Chinese Pharmacopoeia* in 2015 (Zhang et al., 2016; Cao et al., 2017). *A. trifoliata* has medicinal functions mainly in anti-tumor, anti-inflammatory, diuresis, stimulating the menstrual flow, soothing the liver and benefiting the kidney, etc. (Jiang et al., 2012; Cai et al., 2020; Hong et al., 2020; Wang et al., 2020). It also has good curative effect on indigestion and abdominal pain (Xie et al., 2006), and can eliminate toxins in the body and enhance human immunity (An et al., 2016). The sweet and fruity pulp of *A. trifoliata* can be eaten directly or used to make juice, sugar, tea, wine, etc. (Luo et al., 2008; Zhang et al., 2020), and the seeds can be used to extract oil (Zou et al., 2019). Less known fruit species received more popularity recently. They include high content of non-nutritive, nutritive, and bioactive compounds such as flavonoids, phenolics, anthocyanins, phenolic acids, as well as nutritive compounds such as sugars, essential oils, carotenoids, vitamins, and minerals. As a less known fruit species, *A. trifoliata* is a kind of healthy fruit with high nutritional value, excellent medicinal value and good quality (Dogan et al., 2014; Guan et al., 2015; Gecer et al., 2020; Bolaric et al., 2021; Grygorieva et al., 2021).

Due to the edible and medicinal value of the fruit, scholars at home and abroad have carried out some research on *A. trifoliata* in recent years. Li Weiye et al. used a variety of methods to extract *A. trifoliata* seed oil and came up with a better system for seed oil extraction through a series of comparative studies (Li et al., 2021). Tang Chenglin et al. employed several experimental methods to detect and analyze the nutrients in the peel, pulp and seeds and concluded that the fruit had high nutritional value (Tang et al., 2021). Liu Xianglin et al. systematically reviewed and explored the plant propagation technology of fruit-type *A. trifoliata* (Liu et al., 2020). Guo Linxin et al. isolated and identified the chemical constituents of *A. trifoliata*, and tested its antioxidant activity to lay a foundation for the research on the medicinal substances and related mechanisms of *A. trifoliata* (Guo et al., 2017).Huang Rongrong et al. investigated the effect of flash extraction on the yield of *A. trifoliata* seed oil (Huang et al., 2022). Jiang Yongli et al. explored the effects of cell-wall metabolism on *A. trifoliata* fruit cracking (Jiang et al., 2022). Zhang Junming et al. studied the distribution and response to climate change of *A. trifoliata* by the MaxEnt model and ArcGIS (Zhang et al., 2022). Through comparative analysis, we found that the current domestic research on *A. trifoliata* mainly focuses on wild resource investigations (Cai et al., 2020), nutritional components (Guo et al., 2017), and seedling breeding and screening (Liu et al., 2020), etc. What generally lacks in *A. trifoliata* is research on the breeding of new varieties of *A. trifoliata*. Therefore, the selection and breeding of less-seeded (seedless) *A. trifoliata* edible varieties is the premise and basis for promoting the large-scale planting of *A. trifoliata* and industrial development.

With the continuous improvement of people’s pursuit of living standard and healthy life, *A. trifoliata* is classified as the third generation fruit because of its beautiful appearance, sweet taste, rich nutrition and great development value (He, 2018). Although *A. trifoliata* is still a wild plant, it has been gradually loved and sought after by the public as a health-care fruit. The high nutritional value, huge demand and potential industrialization development all show its broad prospects. In this paper, 16 genotypes (clones) of wild *A. trifoliata* germplasm resources collected and domesticated from the Qinba Mountains in the early stage were used to study and analyze the fruit characters and pulp components, in order to provide reference for the basic research and comprehensive utilization of wild *A. trifoliata*, as well as the breeding of new varieties of *A. trifoliata*.

## 2 Materials and methods

### 2.1 Overview of the testing site

The testing site of August Honey Wild Fruit and Tree Research Institute in Huyi District, Xi’an City is located in 108°37’ east longitude, 34°07’ north latitude, 418.8 meters above sea level, and 3.33hm^2^ sandy loam soil. The tested plants are mainly 5-year-old wild varieties and artificially cultivated and colonized in Qinling Mountains, where the planting density is 95-111 plants/667m^2^, the row spacing is 2.0 meters×3.5 meters, and the height is 2.2 meters.

The testing site is situated at the central part of Guanzhong Plain in Shaanxi Province and belongs to the warm temperate zone with semi-humid continental monsoon climate. This area is rich in light, heat and water resources, which is suitable for agricultural production and diversified operations.

### 2.2 Materials

**Figure 1:**
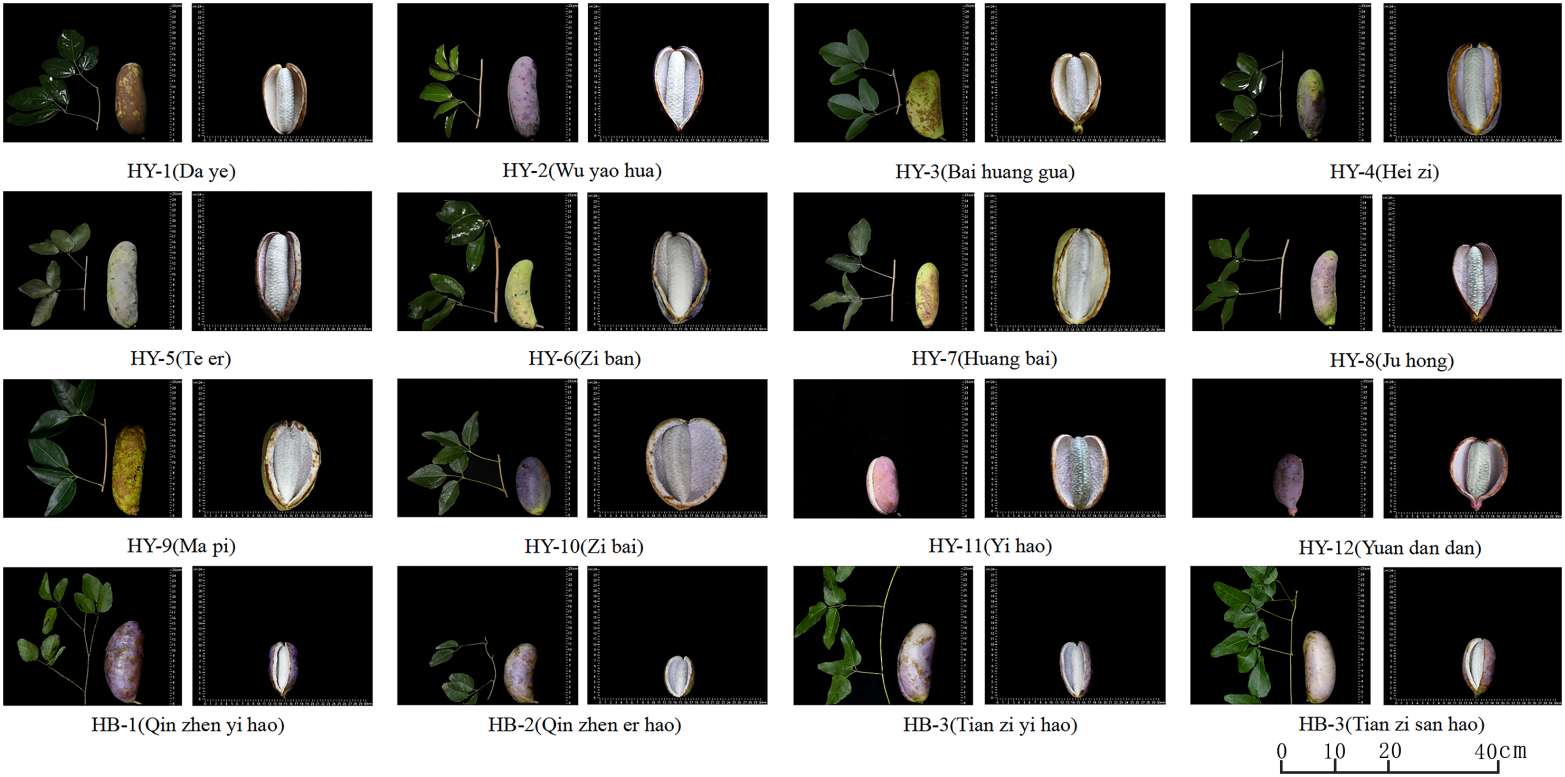
Fruit appearance of 16 A*. trifoliata* genotypes.

The 16 A*. trifoliata* mother plants used in the experiment were all from the Qinba Mountains, 12 of which were from the Qinling Mountains (HY-1, HY-2, HY-3, HY-4, HY-5, HY-6, HY-7, HY-8, HY-9, HY-10, HY-11, HY-12), and the rest 4 were from Bashan Mountains (HB-1, HB-2, HB-3 and HB-4). The soil strength of the testing site is uniform, and the cultivation and management measures of the materials are consistent. Fruits of 16 genotypes were collected in batches during the fruit maturity stage from September to October 2021 (**Figure 1**) and brought back to the laboratory for measurement.

### 2.3 Methods

Fruits were randomly picked from plants of the 16 genotypes. Fruit shape characteristics were determined at stage 1 (Please refer to 2.3 for the division rules of fruit development stages of *A. trifoliata*), and fruit components and pulp nutrient content were measured at stage 3. The measurement indicators are longitudinal diameter, transverse diameter and thickness of the fruit (part of the fruit is flat). The transverse diameter is measured at the widest part and the thickness is measured at the thickest part in the middle of the fruit by a digital vernier caliper. The measurement indicators of fruit composition are single fruit weight, peel weight, pulp weight, seed weight, peel thickness, and number of seeds. A single fruit is firstly weighed and then separated for peel, pulp and seeds to weigh separately by an electronic scale with a precision of 0.01g. The above experiments were replicated for 3 times. The indicators for the nutrient components in pulp are the content of main nutrients (total sugar, total acid, vitamin C, soluble solids, starch), amino acid content, organic acid content and trace element content. The determination methods and standards are in accordance with National Food Safety Standard.

#### 2.3.1 Determination of total sugar

Determined with reference to GB/T 15038-2006 “National Food Safety Standard - Determination of Total Sugar in Food”.

Determination principle: The original sugar in the sample and the sugar produced after hydrolysis are reducible, and it can reduce Fehling’s reagent to generate red cuprous oxide.

#### 2.3.2 Determination of total acid

Determined with reference to GB 12456-2008 “National Food Safety Standard - Determination of Total Acid in Food”.

Determination principle: According to the principle of acid-base neutralization, the acid in the test solution is titrated with alkali, and the titration end point is determined with phenolphthalein as the indicator. Calculate the total acid content in food according to the consumption of lye.

#### 2.3.3 Determination of vitamin C

Determined with reference to GB 5009.86-2016 “National Food Safety Standard - Determination of Ascorbic Acid in Food”.

Determination principle: Using the blue basic dye 2,6-dichlorophenolindophenol standard solution to carry out redox titration on the sample of acid leaching solution containing L(+)-ascorbic acid, 2,6-dichlorophenolindophenol is reduced to colorless. When the titration end point is reached, the excess 2,6-dichlorophenolindophenol appears light red in the acidic medium, and the content of L(+)-ascorbic acid in the sample is calculated by the consumption of 2,6-dichlorophenolindophenol.

#### 2.3.4 Determination of soluble solids

Determined with reference to NY/T 2637-2014 “Determination of Soluble Solid Content in Fruits and Vegetables - Refractometer method”.

Determination principle: Use a refractometer to measure the refractive index of the sample solution, and take notes of the soluble solid content of the sample solution from the display or scale, which is expressed as the mass percentage of sucrose.

#### 2.3.5 Determination of starch

Determined with reference to GB 5009.9-2016 “National Food Safety Standard - Determination of Starch in Food”.

Determination principle: After removing the fat and soluble sugar in the sample, the starch is hydrolyzed into reducing monosaccharides with acid, and then measured as reducing sugars and converted into starch.

#### 2.3.6 Determination of amino acid

Determined with reference to GB 5009.124-2016 “National Food Safety Standard - Determination of Amino Acids in Food”.

Determination principle: The protein in food is hydrolyzed into free amino acids by hydrochloric acid. After being separated by ion exchange column, the free acid reacts with 2,2-Dihydroxyindane-1,3-dione solution, and then the amino acid content is determined by a visible spectrophotometer.

#### 2.3.7 Determination of organic acid

Determined with reference to “National Food Safety Standard - Determination of Organic Acid in Food”.

Determination principle: After directly diluted or extracted with water, the sample is purified by solid-phase extraction with strong anion-exchange columns and separated by a reversed-phase chromatographic column to determine the retention time. External standard method is used for quantification.

#### 2.3.8 Determination of potassium

Determined with reference to GB/T 5009.91-2017 ”National Food Safety Standard - Determination of Potassium and Sodium in Foods”.

Determination principle: After digested, the sample is injected into the atomic absorption spectrometer. After the flame atomization, potassium and sodium absorbed the resonance lines at 766.5 nm and 589.0 nm, respectively. Within a certain concentration range, its absorption value is proportional to the potassium and sodium content, which is quantitatively compared with the standard series.

#### 2.3.9 Determination of calcium

Determined with reference to GB/T 5009.92-2003 “National Food Safety Standard - Determination of Calcium in Foods”.

Determination principle: In the appropriate pH range, calcium forms metal complexes with ethylene diamine tetraacetic acid(EDTA). Using EDTA to titrate, when the equivalence point is reached, the solution takes on the color of the free indicator. Calculate the calcium content according to the amount of EDTA.

#### 2.3.10 Determination of phosphorus

Determined with reference to GB 5009.87-2016 “National Food Safety Standard - Determination of Phosphorus in Foods”.

Determination principle: After the sample is digested, phosphorus combines with ammonium molybdate under acidic conditions to form ammonium phosphomolybdate trihydrate, which is reduced to blue compound molybdenum blue by hydroquinone, sodium sulfite or stannous chloride and hydrazine sulfate. The light absorbancy of molybdenum blue at 660 nm is proportional to the concentration of phosphorus. Use a spectrophotometer to measure the light absorbancy of the sample solution and compare it with the standard series.

#### 2.3.11 Determination of magnesium

Determined with reference to GB 5009.241-2017”National Food Safety Standard - Determination of Magnesium in Foods”.

Determination principle: After digested, the sample is atomized by flame, and the light absorbancy is measured at 285.2 nm. In a certain concentration range, the absorbance value of magnesium is proportional to the magnesium content, and it is quantitatively compared with the standard series.

#### 2.3.12 Determination of zinc

Determined with reference to GB 5009.14-2017”National Food Safety Standard - Determination of Zinc in Foods”.

Determination principle: After digested, the sample is atomized by flame, and the light absorbancy is measured at 213.9 nm. In a certain concentration range, the absorbance value of zinc is proportional to the zinc content, which is quantitatively compared with the standard series.

#### 2.3.13 Determination of copper

Determined with reference to GB 5009.13-2017 “National Food Safety Standard - Determination of Copper in Foods”.

Determination principle: After digested, the sample is atomized in a graphite furnace and the light absorbancy is measured at 324.8 nm. In a certain concentration range, the absorbance value of copper is proportional to the copper content, which is quantitatively compared with the standard series.

#### 2.3.14 Determination of manganese

Determined with reference to GB 5009.242-2017 “National Food Safety Standard - Determination of Manganese in Foods”.

Determination principle: After digested, the sample is injected into the atomic absorption spectrometer. After the flame atomization, the manganese absorbs the resonance line of 279.5 nm. Within a certain concentration range, the absorption value is proportional to the manganese content, which is quantitatively compared with the standard series.

#### 2.3.15 Data analysis

The experimental data were analyzed with Excel and SPSS data processing system.

## 3 Results and analysis

### 3.1 Fruit shape characteristics and components of different genotypes

The flowers of *A. trifoliata* are monoecious, with female flowers in the upper part of the inflorescence and male flowers in the lower part. According to our observation, the development of *A. trifoliata* fruit is firstly the expansion of young fruit. When the fruit stops growing in volume, dehiscent genotypes appear on the curved protrusions of the fruit, then the firmness of the fruit begins to decrease, and the peel takes on the characteristic color and texture of the fruit. The shape of the fruit, the texture and color of the peel vary greatly among different genotypes of *A. trifoliata*. **Table 1** is the fruit shape parameters of different genotypes of *A. trifoliata*. HY-9 has the largest longitudinal diameter of 156.85mm, and HY-1 has the largest transverse diameter and thickness of 62.23mm and 60.26mm, respectively. The longitudinal diameter of the fruit ranges from 104.30mm (HY-11) to 156.85mm (HY-9), the lateral diameter of the fruit ranges from 49.27mm (HY-8) to 62.23mm (HY-1), and the fruit thickness ranges from 46.78mm (HY-8) to 60.26mm (HY-1). The largest difference among the longitudinal diameters of the fruit of different genotypes is 52.55mm, and the transverse diameter (12.96mm) follows. The difference in fruit thickness of different genotypes is less obvious (12.27mm).

**Table 1:** Fruit shape parameters of different genotypes of *A. trifoliata* fruit.

Serial number	Material	Longitudinal diameter/mm	Transverse diameter/mm	Thickness/mm
1	HY-1	149.21 ± 15.95abc	62.23 ± 5.29a	60.26 ± 4.97a
2	HY-2	144.00 ± 20.03abc	57.32 ± 6.04bcd	55.51 ± 7.37bcd
3	HY-3	126.47 ± 22.18de	59.01 ± 7.91abc	53.56 ± 6.38cde
4	HY-4	135.09 ± 9.49cd	59.52 ± 3.35ab	56.04 ± 2.33abc
5	HY-5	141.60 ± 22.63abcd	56.62 ± 5.27bcde	54.93 ± 3.99bcd
6	HY-6	139.44 ± 14.13bcd	53.89 ± 2.92de	49.83 ± 1.85efg
7	HY-7	139.14 ± 12.49bcd	52.63 ± 2.39ef	48.86 ± 2.5fg
8	HY-8	150.62 ± 18.38ab	49.27 ± 2.01f	46.78 ± 1.72g
9	HY-9	156.85 ± 8.63a	59.88 ± 3.19ab	57.95 ± 2.53ab
10	HY-10	115.25 ± 8.76ef	59.00 ± 2.99abc	58.49 ± 2.81ab
11	HY-11	104.30 ± 16.22f	54.92 ± 4.23cde	51.42 ± 4.64def
12	HY-12	——	——	——
13	HB-1	——	——	——
14	HB-2	——	——	——
15	HB-3	——	——	——
16	HB-4	——	——	——
*P*		< 0.05	< 0.05	< 0.05

Data are presented as mean ± SE. Different letters after the values in the same column indicate significant differences among plants with different fruit shape parameters of A. trifoliata (P < 0.05).

Note: The last 5 genotypes were not included in the statistical analysis due to the late picking time, insufficient number of samples, and data vacancies.

ANOVA was performed on the biological characteristics of the fruit. The results showed that there were significant differences in fruit longitudinal diameter, fruit transverse diameter and fruit thickness among different genotypes (*P*<0.01), which indicated that there was obvious diversity in the fruit shape of *A. trifoliata*.

Fruits of different genotypes of *A. trifoliata* are not only different in the shape and color, but also the size and weight. Single fruit weight is an important indicator to measure fruit quality. The *A. trifoliata* fruit contains 3 parts: peel, pulp and seeds whose proportion are directly related to the edibility of the fruit. As can be seen from **Table 2**, the single fruit weight of the 16 genotypes (the average value within the genotype, the same below) ranges from 148.44g (HB-1) to 337.29g (HY-11); the peel weight of a single fruit is between 92.20g (HY-3) and 235.51g (HY-9); the pulp weight of a single fruit is between 34.27g (HB-4) and 95.50g (HY-3); the weight of seeds in a single fruit is between 9.39g (HB-1) and 29.03g (HY-7). The ratio of peel to a single fruit is between 43.58% (HY-3) and 71.92% (HY-9); the ratio of pulp is between 19.04% (HY-9) and 44.80% (HY-3); the ratio of seeds is between 5.58% (HB-3) and 13.12% (HY-7). The number of seeds per fruit is between 143 (HY-8) and 330 (HY-7); the pericarp thickness of a single fruit is between 6.08mm (HY-2) and 12.63mm (HY-9).

**Table 2:** The components and proportions of different genotypes of *A. trifoliata* fruit.

Serial number	Material	Average weight/g				Average ratio/%			Average number of seeds/seed	Average peel thickness/mm
		Total weight of single fruit	Peel weight of single fruit	Single fruit pulp weight	Single fruit seed weight	Pericarp proportion of single fruit	Percentage of single fruit pulp	Percentage of seeds per fruit		
1	HY-1	273.37 ± 59.28b	152.58 ± 32.03bcd	94.65 ± 27.99a	26.54 ± 4.8abc	55.81	34.62	9.71	247 ± 46bc	8.31 ± 1.51ef
2	HY-2	208.15 ± 20.22de	98.15 ± 15.22ef	92.15 ± 7.52a	17.85 ± 3.13ef	47.15	44.27	8.58	179 ± 22defg	6.08 ± 1.16g
3	HY-3	213.19 ± 34.92cde	92.9 ± 22.21f	95.5 ± 16.3a	24.79 ± 8.34abcd	43.58	44.80	11.63	206 ± 23cde	7.81 ± 1.86f
4	HY-4	263.95 ± 60.52bc	171.54 ± 42.27b	67.64 ± 14.79bcd	24.7 ± 7.23abcd	64.99	25.63	9.36	224 ± 47cd	8.88 ± 1.36def
5	HY-5	241.7 ± 68.01bcd	157.65 ± 44.67bc	61.44 ± 16.93cde	22.57 ± 7.79bcde	65.23	25.42	9.34	212 ± 67cd	10.2 ± 1.14bc
6	HY-6	245.02 ± 46.92bcd	152.11 ± 23.77bcd	73.19 ± 22.21bc	19.72 ± 4.88def	62.08	29.87	8.05	179 ± 38defg	9.25 ± 0.84cde
7	HY-7	221.21 ± 45.61cde	112.31 ± 24.31ef	79.88 ± 16.53ab	29.03 ± 5.65a	50.77	36.11	13.12	330 ± 30a	6.43 ± 1.11g
8	HY-8	183.73 ± 53.96ef	121.59 ± 34.72def	46.76 ± 15.58ef	15.38 ± 5.55fg	66.18	25.45	8.37	143 ± 53g	8.29 ± 0.46ef
9	HY-9	327.47 ± 41.89a	235.51 ± 32.53a	62.34 ± 12.13cde	27.47 ± 4.07ab	71.92	19.04	8.39	221 ± 33cd	12.63 ± 1.05a
10	HY-10	175.13 ± 31.67ef	94.17 ± 22.36ef	59.69 ± 11.82cde	21.27 ± 4.13cde	53.77	34.08	12.15	190 ± 41def	6.38 ± 1.53g
11	HY-11	337.29 ± 56.54a	216.74 ± 35.56a	92.32 ± 18.15a	28.34 ± 6.39a	64.26	27.37	8.40	287 ± 75ab	10.49 ± 1.51bc
12	HY-12	183.98 ± 27.39ef	105.27 ± 16.36ef	59.18 ± 12.12cde	19.53 ± 2.37def	57.22	32.17	10.62	193 ± 40def	6.18 ± 0.73g
13	HB-1	148.44 ± 23.19f	104.34 ± 20.92ef	34.72 ± 4.22f	9.39 ± 1.58h	70.29	23.39	6.33	157 ± 35fg	10.76 ± 0.81b
14	HB-2	149.39 ± 29.92f	102 ± 24.61ef	36.76 ± 7.03f	10.64 ± 0.9gh	68.28	24.61	7.12	160 ± 25efg	9.9 ± 1.12bcd
15	HB-3	188.73 ± 48.48ef	127.96 ± 31.41cde	50.24 ± 20.47def	10.53 ± 1.62gh	67.80	26.62	5.58	180 ± 28defg	9.98 ± 1.04bcd
16	HB-4	153.66 ± 28.31f	109.8 ± 19.76ef	34.27 ± 8.82f	9.52 ± 1.37h	71.46	22.30	6.20	149 ± 24fg	10.42 ± 0.63bc
*P*		< 0.05	< 0.05	< 0.05	< 0.05				< 0.05	< 0.05

According to the material source analysis of the 4 genotypes from Bashan Mountains, the average weight of a single fruit, peel and pulp of HB-3 is the largest, which are 188.73g, 127.96g and 50.24g respectively. The average seed weight of HB-2 (10.64g) is the largest. HB-4 has the largest proportion of peel, which is 71.46%. HB-3 has the largest proportion of pulp (26.62%) and the smallest proportion of seeds (5.58%). HB-3 has the highest average number of seeds (180). HB-2 has the least average peel thickness (9.90mm).

Among 12 genotypes from Qinling Mountains, HY-11 has the largest average fruit weight (337.29g), while HY-10 has the least (175.13g), 162.16g lighter than HY-11. HY-9 has the largest average peel weight (235.51g), while HY-3 has the least (92.90g), with a difference of 142.61g. HY-7 has the largest average seed weight (29.03g), while HY-8 has the least (15.38g), with a difference of 13.65g. HY-9 has the largest average ratio of peel to a single fruit (71.92%), while HY-3 has the least (43.58%), with a difference of 28.34%. HY-3 has the largest average ratio of pulp to a single fruit (44.80%), while HY-9 has the least (19.04%), with a difference of 25.76%. HY-6 has the least average ratio of seeds to a single fruit (8.05%), while HY-7 has the most (13.12%), with a difference of 5.07%. HY-8 has the least average number of seeds (143), while HY-7 has the most (330), with a difference of 187 seeds. The average peel thickness of HY-2 is least (6.08mm), and HY-9 is the most (12.63mm), with a difference of 6.55mm. HY-3 has the largest proportion of pulp and higher edible rate, followed by HY-2, which has the thinnest peel and high edible rate.

The fruit characteristic parameters of 16 A*. trifoliata* genotypes were analyzed by variance. There were significant differences in single fruit weight, single fruit peel weight, single fruit pulp weight, single fruit seed weight, single fruit seed number, pericarp thickness and edible rate of *A. trifoliata* fruit of different genotypes (*P*<0.01). It can be seen that in terms of edible rate, there are obvious differences in the fruits of different genotypes of *A. trifoliata*.

### 3.2 Nutritional compositions of fruit pulp of different genotypes

#### 3.2.1 Main nutrient content

Nutritional composition of fruit is an important index and basis for evaluating fruit quality. The determination results of the main nutrients in the pulp of 16 A*. trifoliata* genotypes in Qinba Mountains are shown in **Table 3**. As can be seen from **Table 3**, the total sugar of the 16 A*. trifoliata* genotypes ranges from 10.22g/100g (HY-6) to 19.60g/100g (HY-12) with a difference of 9.38g/100g; the total acid is between 0.06g/100g (HY-3) and 0.25g/100g (HY-5) with a difference of 0.190g/100g; the content of vitamin C is between 9.66mg/100g (HY-11) and 39.80mg/100g (HY-7) with a difference of 30.14mg/100g; the content of soluble solid is between 11.86% (HY-3) and 22.60% (HY-9) with a difference of 10.74%; the starch content is between 1.94g/100g (HY-11) and 9.50g/100g (HY-6) with a difference of 7.56g/100g. In general, the nutrient content of each genotype was not much different.

**Table 3:** The content of main chemical components in the pulp of different genotypes of *A. trifoliata* fruit.

Serial number	Material	Total sugar (g/100g)	Total acid (g/100g)	Vitamin C (mg/100g)	Soluble solids (%)	Starch (g/100g)	Coefficient of variation
1	HY-1	16.67 ± 0.08bc	0.12 ± 0.01h	24.21 ± 0.01h	19.08	8.45 ± 0.01b	1.01
2	HY-2	17.07 ± 0.02b	0.10 ± 0.00i	36.25 ± 0.05b	20.39	3.55 ± 0.03h	1.19
3	HY-3	10.79 ± 0.07h	0.06 ± 0.00j	30.29 ± 0.04e	11.86	3.39 ± 0.01h	1.10
4	HY-4	15.70 ± 0.01c	0.14 ± 0.01e	29.66 ± 0.04f	18.60	8.15 ± 0.02c	1.01
5	HY-5	13.15 ± 0.01fg	0.25 ± 0.00a	33.80 ± 0.22d	18.10	8.49 ± 0.03b	0.99
6	HY-6	10.22 ± 0.01h	0.13 ± 0.01f	35.91 ± 0.23c	14.70	9.5 ± 0.04a	0.95
7	HY-7	14.36 ± 0.07de	0.18 ± 0.01b	39.80 ± 0.2a	18.50	4.37 ± 0.01g	1.13
8	HY-8	17.55 ± 0.17b	0.17 ± 0.00c	27.90 ± 0.24g	22.00	4.28 ± 0.01fg	1.17
9	HY-9	19.03 ± 0.01a	0.10 ± 0.01i	23.76 ± 0.06i	22.60	5.14 ± 0.03e	1.14
10	HY-10	13.72 ± 0.01bc	0.25 ± 0.00a	29.63 ± 0.08f	18.00	2.86 ± 0.01i	1.19
11	HY-11	12.84 ± 0.02g	0.12 ± 0.00g	9.66 ± 0.01o	13.56	1.94 ± 0.03j	1.21
12	HY-12	19.60 ± 0.03a	0.11 ± 0.00h	22.31 ± 0.17j	21.57	3.09 ± 0.04i	1.21
13	HB-1	14.48 ± 0.04d	0.14 ± 0.00e	20.26 ± 0.01l	16.10	5.96 ± 0.02d	1.04
14	HB-2	13.26 ± 0.05efg	0.14 ± 0.00e	18.29 ± 0.02n	15.20	4.38 ± 0.09f	1.09
15	HB-3	12.40 ± 0.01g	0.14 ± 0.00e	20.93 ± 0.03k	18.20	5.03 ± 0.01e	1.11
16	HB-4	14.05 ± 0.06def	0.16 ± 0.00d	19.72 ± 0.12m	18.70	6.02 ± 0.03d	1.07
Average value		14.68 ± 0.04	0.14 ± 0.00	26.40 ± 0.09	17.95	5.29 ± 0.02	1.09
*P*		< 0.05	< 0.05	< 0.05	< 0.05	< 0.05	


Data are presented as mean ± SE (n = 3). Different letters after the values in the same column indicate significant differences among plants with the content of main chemical components in the pulp of different genotypes of A. trifoliata fruit (P < 0.05).

It can be seen from **Table 3** that, the main chemical components in *A. trifoliata* pulp include total sugar, total acid, vitamin C, soluble solids and starch. Through analysis of the main nutrients in pulp of 16 A*. trifoliata* genotypes, the difference in the content of vitamin C shows the highest value (30.14mg/100g); the difference in total acid content shows the lowest value (0.190g/100g). In addition, the ratio of total sugar to total acid content is relatively high, which can increase the flavor of the fruit and is one of the parameters of the sweet and glutinous taste of the fruit. The content of vitamin C in HY-7 pulp reaches 39.80mg/100g, which is lower than that of kiwi (95.5mg/100g), orange (64.1mg/100g), grape (43.2mg/100g), but higher than that of tangerinr (38.5mg/100g) and strawberry (37.3mg/100g) (Zou, 2021). It shows that the fruit of *A. trifoliata* is rich in chemical components and has high nutritional value. However, this is relatively different from the research results of Liu Lunpei et al. (vitamin C content is 108mg/100g) and Wan Mingchang et al. (vitamin C content is 0.34mg/100g), thus further testing is required. The average starch content is 5.29g/100g, which is relatively high and quite different from the research results of Wan Mingchang et al., thus further testing is required as well.

By comparing the coefficient of variation (CV) of the 16 genotypes, it is found that the CV of HY-11 and HY-12 shows the highest (1.21), indicating that the degree of variation of these two genotypes is higher than that of the other 14 genotypes, but not obvious. The CV of HY-6 is 0.95, the lowest among the 16 genotypes.

#### 3.2.2 Amino acid content

Amino acids are essential to maintain the homeostasis of life and plant growth and reproduction. The determination results of amino acid content in pulp of the 16 A*. trifoliata* genotypes in Qinba Mountains are shown in **Table 4**. As can be seen from it, HY-1 has the most types of amino acids in pulp up to 17, including 7 essential amino acids for human body (Lys, Phe, Met, Thr, Ile, Leu and Val) and other 10 amino acids (such as Asp, Glu, etc.); HY-11 has the least types of amino acids (13 types).

**Table 4:** The content of essential amino acids in the pulp of different genotypes of *A. trifoliata* fruit(mg/100g).

Serial number	Material	Thr	Val	Met	Ile	Leu	Phe	Lys	The total content and proportion of essential amino acids		Total amino acid content
									The content	The proportion(%)	
1	HY-1	30 ± 5a	20 ± 10	10 ± 5a	20 ± 5a	30 ± 5a	20 ± 10	20 ± 10	160	20.00	800
2	HY-2	20 ± 5ab	20 ± 0	0 ± 0b	20 ± 10a	40 ± 10a	20 ± 5	30 ± 5	150	25.86	580
3	HY-3	20 ± 10ab	20 ± 10	0 ± 0b	20 ± 10a	30 ± 0a	20 ± 15	30 ± 5	140	35.00	400
4	HY-4	20 ± 0ab	20 ± 10	10 ± 5a	10 ± 5ab	30 ± 10a	20 ± 5	20 ± 10	130	38.24	340
5	HY-5	10 ± 5b	20 ± 0	0 ± 0b	10 ± 5ab	30 ± 10a	10 ± 5	20 ± 0	100	14.29	700
6	HY-6	10 ± 0b	20 ± 10	0 ± 0b	20 ± 5a	30 ± 5a	20 ± 5	30 ± 5	130	37.14	350
7	HY-7	20 ± 5ab	20 ± 0	0 ± 0b	20 ± 5a	40 ± 5a	20 ± 0	20 ± 10	140	37.84	370
8	HY-8	20 ± 10ab	20 ± 10	0 ± 0b	10 ± 10ab	30 ± 10a	10 ± 0	20 ± 5	110	18.64	590
9	HY-9	30 ± 10a	20 ± 0	0 ± 0b	20 ± 10a	40 ± 0a	20 ± 5	30 ± 10	160	18.60	860
10	HY-10	10 ± 5b	20 ± 0	0 ± 0b	10 ± 0ab	30 ± 5a	10 ± 5	20 ± 5	100	32.26	310
11	HY-11	10 ± 5b	10 ± 0	0 ± 0b	0 ± 0b	10 ± 0b	10 ± 5	10 ± 10	50	27.78	180
12	HY-12	20 ± 0ab	20 ± 10	0 ± 0b	20 ± 5a	40 ± 10a	20 ± 10	20 ± 0	140	38.89	360
13	HB-1	10 ± 5b	20 ± 10	0 ± 0b	10 ± 0ab	30 ± 10a	10 ± 0	20 ± 5	100	29.41	340
14	HB-2	10 ± 10b	10 ± 0	0 ± 0b	10 ± 5ab	30 ± 0a	10 ± 10	20 ± 15	90	30.00	300
15	HB-3	10 ± 0b	20 ± 10	0 ± 0b	10 ± 10ab	30 ± 5a	10 ± 5	20 ± 10	100	27.03	370
16	HB-4	10 ± 5b	20 ± 10	0 ± 0b	10 ± 5ab	30 ± 10a	10 ± 5	20 ± 5	100	30.30	330
*P*		< 0.05	——	< 0.05	< 0.05	< 0.05	——	——			

Data are presented as mean ± SE (n = 3). Different letters after the values in the same column indicate significant differences among plants with the content of essential amino acids in the pulp of different genotypes of A. trifoliata fruit (P < 0.05).

Note: The abbreviations for amino acids are as follows: Lysine (Lys), Phenylalanine (Phe), Methionine (Met), L-Threonine (Thr), l-isoleucine (Ile), Leucine(Leu) amd Valine(Val).

According to **Tables 4**, **5**, HY-9 has the highest total amino acid in pulp (860mg/100g), and HY-11 has the least total amino acid (180mg/100g). The total amino acid content in pulp of the 16 A*. trifoliata* genotypes from high to low is as follows, HY-8, HY-1, HY-7, HY-6, HY-9, HY-2, HY-4, HB-2, HB-1, HY-12, HY-3, HB-3, HY-11, HY-5, HB-4 and HY-10.

**Table 5:** The content of other amino acids in the pulp of different genotypes of *A. trifoliata* fruit(mg/100g).

Serial number	Material	Asp	Ser	Glu	Pro	Gly	Ala	Cys	Tyr	His	Arg	Total content and proportion of other amino acids		Total amino acid content
												The content	The proportion(%)	
1	HY-1	70 ± 20a	30 ± 5a	80 ± 15a	300 ± 10c	30 ± 10	20 ± 0	20 ± 5ab	10 ± 0	20 ± 5a	60 ± 10ab	640	80.00	800
2	HY-2	60 ± 10ab	30 ± 0a	50 ± 10bc	180 ± 20d	20 ± 10	10 ± 0	20 ± 5ab	10 ± 0	30 ± 10a	20 ± 10ef	430	74.14	580
3	HY-3	50 ± 10bc	20 ± 10ab	80 ± 10a	0 ± 0e	20 ± 0	10 ± 10	20 ± 0ab	10 ± 10	30 ± 10a	20 ± 5ef	260	65.00	400
4	HY-4	40 ± 5cd	20 ± 5ab	50 ± 10bc	0 ± 0e	20 ± 5	10 ± 5	20 ± 0ab	10 ± 0	20 ± 10a	20 ± 10ef	210	61.76	340
5	HY-5	30 ± 5d	20 ± 10ab	70 ± 5ab	390 ± 10b	20 ± 15	10 ± 0	20 ± 0ab	10 ± 5	20 ± 10a	10 ± 10f	600	85.71	700
6	HY-6	40 ± 5cd	20 ± 0ab	50 ± 10bc	0 ± 0e	20 ± 10	10 ± 10	20 ± 10ab	10 ± 0	30 ± 5a	20 ± 5ef	220	62.86	350
7	HY-7	40 ± 10cd	30 ± 10a	60 ± 10abc	0 ± 0e	20 ± 0	10 ± 5	20 ± 0ab	10 ± 10	30 ± 10a	10 ± 5f	230	62.16	370
8	HY-8	40 ± 10cd	20 ± 10ab	50 ± 5bc	290 ± 10c	20 ± 10	10 ± 0	10 ± 10bc	10 ± 0	20 ± 5a	10 ± 5f	480	81.36	590
9	HY-9	70 ± 5a	30 ± 5a	50 ± 15bc	420 ± 40a	30 ± 10	20 ± 10	20 ± 0ab	10 ± 5	20 ± 5a	30 ± 0de	700	81.40	860
10	HY-10	40 ± 5cd	20 ± 5ab	50 ± 5bc	0 ± 0e	20 ± 10	10 ± 0	20 ± 5ab	10 ± 0	30 ± 5a	10 ± 5f	210	67.74	310
11	HY-11	20 ± 5e	10 ± 0b	40 ± 10c	0 ± 0e	10 ± 0	10 ± 5	30 ± 5a	0 ± 0	0 ± 0b	10 ± 0f	130	72.22	180
12	HY-12	50 ± 10bc	20 ± 0ab	60 ± 10abc	0 ± 0e	20 ± 5	10 ± 10	0 ± 0c	10 ± 5	20 ± 5a	30 ± 5de	220	61.11	360
13	HB-1	40 ± 0cd	20 ± 5ab	50 ± 10bc	0 ± 0e	20 ± 5	10 ± 10	20 ± 0ab	10 ± 0	30 ± 5a	40 ± 10cd	240	70.59	340
14	HB-2	30 ± 5d	10 ± 10b	40 ± 10c	0 ± 0e	20 ± 0	10 ± 0	20 ± 10ab	10 ± 10	20 ± 10a	50 ± 10bc	210	70.00	300
15	HB-3	40 ± 10cd	20 ± 10ab	50 ± 10bc	0 ± 0e	20 ± 5	10 ± 5	20 ± 10ab	10 ± 10	30 ± 15a	70 ± 10a	270	72.97	370
16	HB-4	30 ± 10d	20 ± 0ab	50 ± 20bc	0 ± 0e	20 ± 0	10 ± 0	20 ± 10ab	10 ± 0	20 ± 15a	50 ± 10bc	230	69.70	330
*P*		< 0.05	< 0.05	< 0.05	< 0.05	——	——	< 0.05	——	< 0.05	< 0.05			

Data are presented as mean ± SE (n = 3). Different letters after the values in the same column indicate significant differences among plants with the content of other amino acids in the pulp of different genotypes of A. trifoliata fruit (P < 0.05).

Note: The abbreviations for amino acids are as follows: Aspartic acid (Asp), Serine (Ser), Glutamic acid (Glu), Proline (Pro), Glycine (Gly), Alanine (Ala), Cystine (Cys), Tyrosine (Tyr), Histidine (His) and Arginine (Arg).

As can be seen from **Table 4**, HY-1 and HY-9 have the highest total content of essential amino acids in the pulp of different genotypes of *A. trifoliata* (160mg/100g), accounting for 20.00% of the total amino acid content; HY-11 has the least (50mg/100g), accounting for 27.78%. Among them, HY-9 and HY-1 have the highest threonine (30mg/100g). HY-11 and HB-2 have the least valine (10mg/100g), while the valine content of other genotypes is 20mg/100g. The content of methionine is the least (10mg/100g), only existing in HY-1 and HY-4. All the other genotypes but HY-11 contain isoleucine, and HY-11 has the least leucine and lysine (both 10mg/100g). All genotypes contain phenylalanine with small difference, HY-11 has 10mg/100g and HY-1 has 20mg/100g. The total content of essential amino acids in HY-4 is 130mg/100g, accounting for 38.24% of the total amino acid; HY-5 contains the least essential amino acids, accounting for only 14.29%.

According to **Table 5**, HY-9 has the most content of other amino acids in the pulp of different genotypes of *A. trifoliata* (700mg/100), accounting for 81.40% of the total amino acid content. HY-11 has the least content (130mg/100g), accounting for 72.22%. HY-1 and HY-9 have the most aspartic acid (70mg/100g), and HY-11 has the least (20mg/100g). HY-1 and HY-2 have the highest content of serine (30mg/100g), and HY-1 and HY-3 have the highest content of glutamic acid (80mg/100g). The content of proline detected in HY-9 is the highest (420mg/100g), followed by HY-5 with a content of 390mg/100g. HY-3, HY-4 and other genotypes do not contain proline, and the difference is obvious. HY-1 has the most glycine, and HY-11 has the least. The glycine content of other genotypes is 20mg/100g. HY-1 and HY-9 have 20mg/100g alanine, and the alanine content of other genotypes is 10mg/100g. HY-11 has the highest content of cystine (30mg/100g), and no cystine is detected in HY-12. HY-11 does not contain tyrosine, and the content of tyrosine in other genotypes is 10mg/100g. No histidine is detected in HY-11, but the content of histidine in 7 genotypes including HY-2 is the highest (30 mg/100g). HB-3 has the most arginine (70mg/100g), and 5 genotypes including HY-5 have the least arginine (10mg/100g).

Most amino acids have a sense of taste and play a role in sourness, sweetness, bitterness, astringency and other tastes in food. According Tang Chenglin et al., amino acids are divided into four characteristic amino acids (Wan et al., 2008; Tang et al., 2021), namely umami amino acids, sweet amino acids, aromatic amino acids, and medicinal amino acids.

According to **Table 6**, 17 amino acids are contained in the pulp of different genotypes of *A. trifoliata.* HY-9 has the highest umami amino acid content (650mg/100g); HY-1 and HY-9 have the highest content of sweet amino acids (110mg/100g). Aromatic amino acids mainly include tyrosine and phenylalanine. Among the 16 genotypes, their content is relatively low, and the difference is small (20~30mg/100g). HY-1 has the highest content of medicinal amino acids (350mg/100g). In addition, there are other amino acids that have an impact on bitterness, such as valine (mostly 20mg/100g) in HY-1.

**Table 6:** The content of characteristic amino acids in the pulp of different genotypes of *A. trifoliata* fruit(mg/100g).

Serial number	Material	Umami amino acid (Glu, Asp, Thr, Ser, Pro, Gly and Ala)	Sweet amino acid (Ala, Gly, Thr and Ser)	Aromatic amino acids (Tyr, Phe)	Medicinal amino acids (Asp, Glu, Arg, Gly, Ile, Leu, Phe, Lys and Met)
1	HY-1	560	110	30	350
2	HY-2	370	80	30	260
3	HY-3	200	70	30	270
4	HY-4	160	70	30	220
5	HY-5	550	60	20	200
6	HY-6	150	60	30	230
7	HY-7	180	80	30	230
8	HY-8	450	70	20	190
9	HY-9	650	110	30	290
10	HY-10	150	60	20	190
11	HY-11	100	40	10	110
12	HY-12	180	70	30	260
13	HB-1	150	60	20	220
14	HB-2	120	50	20	210
15	HB-3	150	60	20	250
16	HB-4	140	60	20	220
Total content		270	70	30	230

#### 3.2.3 Organic acid content

Organic acids are the main flavor nutrients in fruits. **Table 7** shows the measurement results of organic acid content in pulp of the 16 A*. trifoliata* genotypes in Qinba Mountains. As can be seen from **Table 7**, there are mainly four organic acids in pulp, namely malic acid, lactic acid, citric acid and fumaric acid. Four of the 16 genotypes contain four organic acids, namely HY-3, HY-4, HY-6 and HY-7. Seven genotypes contain three organic acids, namely HY-1, HY-2, HY-3, HB-8, HY-9, HY-11 and HY-12. The ones containing only two organic acids are HY-1, HB-2, HB-4, HB-5 and HY-10. Among them, HB-2 and HY-5 have the highest content of malic acid (1.40g/kg), while HY-4 has the least (0.63g/kg), with a difference of 0.77g/kg. HB-2 and HB-4 have the most lactic acid (9.20g/kg), and the ones without lactic acid in the pulp are HY-8, HY-11 and HY-12 respectively. HY-6 has the highest content of citric acid, while 8 genotypes (HY-1, HY-2, HY-9, HY-10, HB-1, HB-2, HB-3, HB-4) have no citric acid detected. HY-3 has the most fumaric acid (0.062g/kg), but 5 genotypes(HY-5, HY-10, HB-1, HB-2, HB-4) have no fumaric acid detected.

**Table 7:** The content of organic acid in the pulp of different genotypes of *A. trifoliata* fruit(g/kg).

Serial number	Material	Malic acid	Lactic acid	Citric acid	Fumaric acid	Coefficient of variation
1	HY-1	1.10 ± 0.06d	3.10 ± 0.06f	0.00 ± 0.00h	0.0300 ± 0.0004c	1.38
2	HY-2	1.30 ± 0.00bc	1.10 ± 0.06g	0.00 ± 0.00h	0.0300 ± 0.0005c	1.13
3	HY-3	0.69 ± 0.00g	4.20 ± 0.06e	0.47 ± 0.00f	0.062 ± 0.0017a	1.41
4	HY-4	0.63 ± 0.00g	0.84 ± 0.06h	0.60 ± 0.01e	0.0090 ± 0.0001f	0.69
5	HY-5	1.40 ± 0.00a	0.88 ± 0.06h	0.48 ± 0.01f	0.0000 ± 0.0000i	0.86
6	HY-6	1.00 ± 0.06e	1.20 ± 0.00g	0.94 ± 0.02a	0.0420 ± 0.0006b	0.65
7	HY-7	0.81 ± 0.00f	0.60 ± 0.00i	0.39 ± 0.00g	0.0017 ± 0.0001h	0.77
8	HY-8	0.82 ± 0.00f	0.00 ± 0.00j	0.93 ± 0.00b	0.0089 ± 0.0004f	1.15
9	HY-9	0.80 ± 0.00f	5.00 ± 0.1d	0.00 ± 0.00h	0.0220 ± 0.0009d	1.64
10	HY-10	1.00 ± 0.06e	1.10 ± 0.06g	0.00 ± 0.00h	0.0000 ± 0.0000i	1.16
11	HY-11	1.20 ± 0.00c	0.00 ± 0.00j	0.83 ± 0.00c	0.0055 ± 0.0000g	1.19
12	HY-12	1.10 ± 0.06d	0.00 ± 0.00j	0.66 ± 0.02d	0.0086 ± 0.0004f	1.21
13	HB-1	1.30 ± 0.06b	9.00 ± 0.15b	0.00 ± 0.00h	0.0000 ± 0.0000i	1.68
14	HB-2	1.40 ± 0.06ab	9.20 ± 0.06a	0.00 ± 0.00h	0.0000 ± 0.0000i	1.67
15	HB-3	1.00 ± 0.06e	8.70 ± 0.06c	0.00 ± 0.00h	0.018 ± 0.0006e	1.73
16	HB-4	0.85 ± 0.06f	9.20 ± 0.00a	0.00 ± 0.00h	0.0000 ± 0.0000i	1.78
Average value		1.03 ± 0.03	3.38 ± 0.05	0.33 ± 0.00	0.0149 ± 0.0000	1.28
*P*		< 0.05	< 0.05	< 0.05	< 0.05	

Data are presented as mean ± SE (n = 3). Different letters after the values in the same column indicate significant differences among plants with the content of organic acid in the pulp of different genotypes of A. trifoliata fruit (P < 0.05).

By CV of variation of the 16 genotypes in **Table 7**, it was found that the CV of HB-4 reached 1.78, ranking first among the 16 genotypes, indicating that the degree of variation of HB-4 was greater than that of the other 15 genotypes. The CV of HY-6 was the lowest among the 16 genotypes (0.65), indicating that the degree of variation of HY-6 was lower than that of the other 15 genotypes.

#### 3.2.4 Mineral content

Minerals are of great significance to maintain the normal life activities of human. **Table 8** shows the determination results of minerals in pulp of the 16 A*. trifoliata* genotypes in Qinba Mountains. It can be seen from **Table 8** that there are 8 mineral elements (4 macro elements, namely potassium, calcium, phosphorus, magnesium and 4 micro elements, namely zinc, iron, copper, and manganese) in the pulp of the 16 genotypes. Among them, the content of potassium is between 1.33g/kg (HY-9) and 2.64mg/kg (HY-1), with a difference of 1.31mg/kg and an average content of 1.83mg/kg; the calcium content is between 0.17g/kg (HY-6) and 0.34g/kg (HB-4), with a difference of 0.17g/kg and an average content of 0.23g/kg; the phosphorus content is between 0.15g/kg (HY-7) and 0.41g/kg (HY-1), with a difference of 0.26g/kg and an average content of 0.29g/kg; the magnesium content is between 0.08g/kg (HY-7) and 0.33g/kg (HB-4), with a difference of 0.25g/kg and an average content of 0.21g/kg; the zinc content is between 0.57mg/kg (HY-7) and 5.67mg/kg (HB-2), with a difference of 5.10mg/kg and an average content of 2.23mg/kg; the iron content is between 1.04mg/kg (HY-7) and 4.32mg/kg (HY-6), with a difference of 3.28mg/kg and an average content of 2.29mg/kg; the copper content is between 0.62mg/kg (HY-10) and 2.16mg/kg (HB-1), with a difference of 1.54mg/kg and an average content of 1.37mg/kg; the manganese content is from 2.18mg/kg (HY-8) to 8.39mg/kg (HY-9), with a difference of 6.21mg/kg and an average content of 5.52mg/kg.

**Table 8:** The content of minerals in the pulp of different genotypes of *A. trifoliata* fruit.

Serial number	Material	Potassium (g/kg)	Calcium(g/kg)	Phosphorus (g/kg)	Magnesium (g/kg)	Zinc (mg/kg)	Iron (mg/kg)	Copper (mg/kg)	Manganese (mg/kg)
1	HY-1	2.64 ± 0.05a	0.19 ± 0.00ij	0.41 ± 0.01a	0.21 ± 0.00f	1.75 ± 0.02g	2.00 ± 0.02e	2.00 ± 0.02b	6.91 ± 0.06de
2	HY-2	2.02 ± 0.05c	0.20 ± 0.00hi	0.36 ± 0.01bc	0.20 ± 0.01g	1.29 ± 0.01h	1.68 ± 0.01i	0.84 ± 0.04ij	3.90 ± 0.03ij
3	HY-3	1.82 ± 0.07de	0.23 ± 0.00efg	0.25 ± 0.00g	0.18 ± 0.01h	2.4 ± 0.10d	3.09 ± 0.04b	1.74 ± 0.07c	6.98 ± 0.12d
4	HY-4	1.82 ± 0.06d	0.22 ± 0.01fgh	0.33 ± 0.01d	0.22 ± 0.01ef	1.13 ± 0.04i	2.30 ± 0.09d	1.34 ± 0.04e	4.13 ± 0.16i
5	HY-5	1.7 ± 0.02f	0.26 ± 0.00cd	0.28 ± 0.01f	0.18 ± 0.00h	0.73 ± 0.01k	1.73 ± 0.02hi	0.79 ± 0.01j	3.22 ± 0.03k
6	HY-6	2.35 ± 0.02b	0.17 ± 0.00j	0.37 ± 0.00b	0.20 ± 0.00g	0.93 ± 0.01j	4.32 ± 0.05a	1.61 ± 0.01d	3.90 ± 0.03ij
7	HY-7	1.52 ± 0.06g	0.19 ± 0.01ij	0.15 ± 0.01j	0.08 ± 0.00j	0.57 ± 0.01l	1.04 ± 0.06k	0.86 ± 0.03i	7.35 ± 0.30c
8	HY-8	2.05 ± 0.06c	0.25 ± 0.01de	0.35 ± 0.01c	0.28 ± 0.01c	1.96 ± 0.07f	1.93 ± 0.07e	2.03 ± 0.07b	2.18 ± 0.11k
9	HY-9	1.33 ± 0.00h	0.23 ± 0.01fg	0.21 ± 0.01hi	0.18 ± 0.00h	1.29 ± 0.01h	4.26 ± 0.01a	0.98 ± 0.01h	8.39 ± 0.03a
10	HY-10	1.50 ± 0.04g	0.20 ± 0.03gh	0.30 ± 0.01e	0.15 ± 0.00i	0.62 ± 0.00l	1.50 ± 0.08j	0.62 ± 0.02k	6.64 ± 0.14e
11	HY-11	1.79 ± 0.03ef	0.28 ± 0.01c	0.23 ± 0.01h	0.18 ± 0.01h	3.54 ± 0.06c	1.79 ± 0.04gh	1.38 ± 0.03e	4.93 ± 0.11h
12	HY-12	2.08 ± 0.03c	0.22 ± 0.00fgh	0.34 ± 0.01d	0.26 ± 0.01d	2.15 ± 0.03e	2.02 ± 0.03e	1.23 ± 0.02f	3.67 ± 0.07j
13	HB-1	1.75 ± 0.02ef	0.24 ± 0.01def	0.25 ± 0.01g	0.22 ± 0.01e	5.6 ± 0.08a	1.56 ± 0.02j	2.16 ± 0.00a	7.67 ± 0.33b
14	HB-2	1.70 ± 0.01f	0.31 ± 0.01b	0.28 ± 0.01f	0.30 ± 0.00b	5.67 ± 0.02a	1.85 ± 0.01g	1.69 ± 0.01c	7.52 ± 0.01bc
15	HB-3	1.46 ± 0.04g	0.2 ± 0.01hi	0.21 ± 0.01i	0.2 ± 0.01g	2.41 ± 0.05d	3.11 ± 0.07b	1.1 ± 0.03g	5.28 ± 0.15g
16	HB-4	1.76 ± 0.02ef	0.34 ± 0.01a	0.27 ± 0.01f	0.33 ± 0.00a	3.67 ± 0.02b	2.43 ± 0.14c	1.6 ± 0.01d	5.68 ± 0.03f
Average value		1.83 ± 0.04	0.23 ± 0.01	0.29 ± 0.01	0.21 ± 0.01	2.23 ± 2.25	2.29 ± 0.05	1.37 ± 0.03	5.52 ± 0.11
*P*		< 0.05	< 0.05	< 0.05	< 0.05	——	< 0.05	< 0.05	< 0.05


Data are presented as mean ± SE (n = 3). Different letters after the values in the same column indicate significant differences among plants the content of minerals in the pulp of different genotypes of A. trifoliata fruit (P < 0.05).

### 3.3 Changes in nutrient composition of fruit pulp at different developmental stages

**Figure 2:**
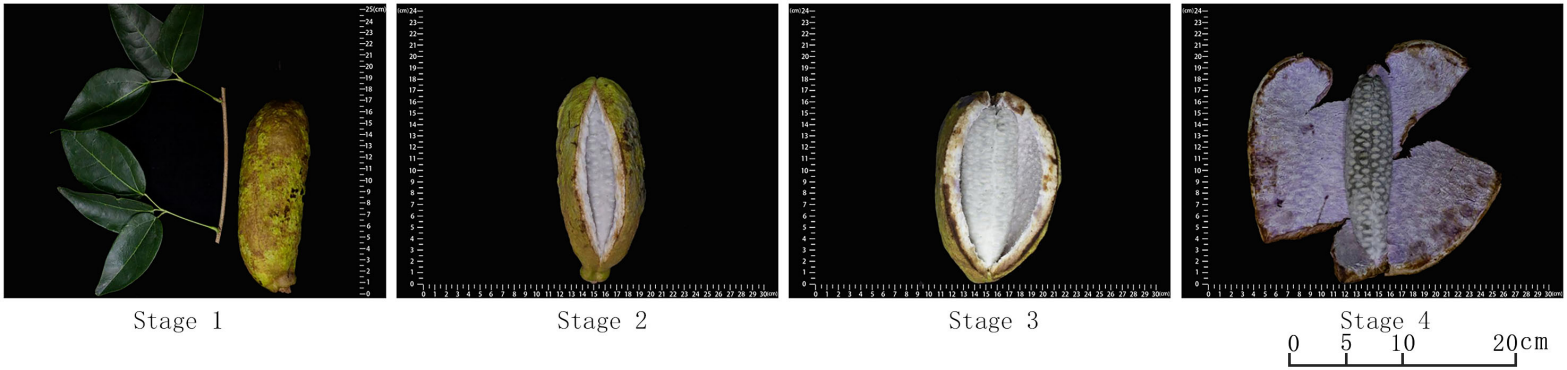
Four stages of fruit development of *A. trifoliata* fruit (taking HY-9 material as an example).

According to years of field observation, it is found that the apparent characteristics of *A. trifoliata* fruit change significantly during the ripening process, which are manifested in various aspects such as fruit size, shape, hardness, color, texture, etc. Compared with other fruits, there is a cracking during the ripening of *A. trifoliata* fruit. HY-9 was chosen as the experimental material for this part because its maturation time is basically the same as other genotypes (the fruits ripen from the end of September to the beginning of October every year), the peel is green with brown markings, the surface is rough, uneven, the fruit size is uniform, and the taste is good. In addition, at the mature stage, the fruit changes are obvious, and it is easy to distinguish four stages, which is the same as other varieties, so HY-9 can well represent the characteristics of all genotypes. Taking HY-9 as an example, the later growth and development of fruit is divided into the following four stages (as shown in **Figure 2**), and the characteristics of each stage are as follows. In stage 1, the fruit has completed longitudinal extension and lateral expansion, the peel has a specific shape, color and texture with obvious back stitches, and is not cracked. In stage 2, the fruit is slightly cracked along the dorsal suture, the pulp is milky white, firm and opaque. In stage 3, the fruit takes on larger crack, and the pulp is soft and translucent. At this time, the pulp tastes the best, thus this stage is the best edible period for *A. trifoliata* fruit. In stage 4, the fruit is fully cracked and the pulp begins to decay.

#### 3.3.1 Changes of main nutrients in fruits at different developmental stages

**Figure 3:**
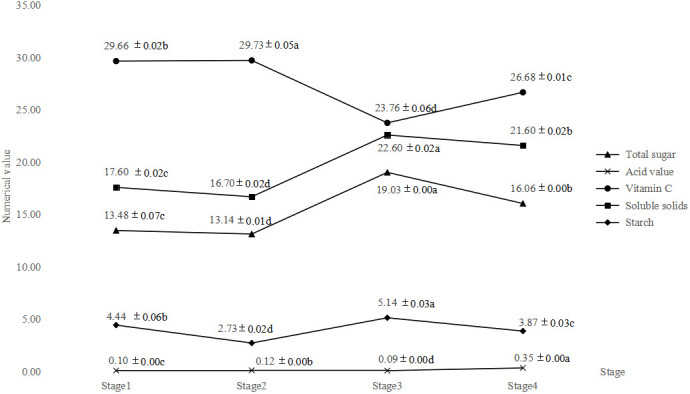
Changes of main nutrient components in the pulp at different developmental stages of fruit.

**Table 9** shows the determination results of the main nutrient components in the pulp at different developmental stages of the fruit. It can be seen from **Table 9** and **Figure 3** that during the growth and development of *A. trifoliata* fruit, with the maturity of the fruit, the total sugar content and soluble solid content in the pulp show an upward trend and reach the peak at stage 3, which are 19.03g/100g and 22.60%, respectively. The results confirm the fact that *A. trifoliata* fruit had the best taste in stage 3. After stage 3, the total sugar content and soluble solid content of the fruit begin to decrease. The total acid content of the fruit does not change much from stage 1 to stage 3 and reaches the minimum value (0.09g/100g) in stage 3, which may be the reason why the *A. trifoliata* fruit does not taste sour. But in stage 4, the total acid content rises rapidly to 0.35g/100g, which may be due to the rancidity of the pulp with the decay of the fruit. The content of vitamin C shows an upward trend before stage 3 with the ripening of the fruit, decreases greatly in stage 3, and then increases slightly. The starch content shows a trend of first decreasing, then increasing, and then decreasing again.

**Table 9:** The content of main nutrients in the pulp at different developmental stages of fruit.

Stage	Total sugar(g/100g)	Total acid(g/100g)	Vitamin C(mg/100g)	Soluble solids(%)	Starch(g/100g)
1	13.48 ± 0.07c	0.1 ± 0c	29.66 ± 0.02b	17.60 ± 0.02c	4.44 ± 0.06b
2	13.14 ± 0.01d	0.12 ± 0b	29.73 ± 0.05a	16.70 ± 0.02d	2.73 ± 0.02d
3	19.03 ± 0.00a	0.09 ± 0.00d	23.76 ± 0.06d	22.60 ± 0.02a	5.14 ± 0.03a
4	16.06 ± 0.00b	0.35 ± 0.00a	26.68 ± 0.01c	21.60 ± 0.02b	3.87 ± 0.03c
*P*	< 0.05	< 0.05	< 0.05	< 0.05	< 0.05


Data are presented as mean ± SE (n = 3). Different letters after the values in the same column indicate significant differences among plants the content of main nutrients in the pulp at different developmental stages of fruit (P < 0.05).

ANOVA was performed on the parameters of main nutrients of the pulp in different developmental stages of HY-9 fruit. The results showed that there were significant differences in total sugar, total acid, vitamin C, soluble solids and starch in different developmental stages of HY-9 fruit (*P*<0.01), which indicated that there was obvious diversity in the main nutrients of HY-9 fruit pulp at different developmental stages.

In the process of fruit ripening, the content of nutrients in the pulp is constantly changing, which is the result of the continuous synthesis, decomposition and transformation of the internal substances in the pulp. This process is also affected by external environmental conditions such as light and temperature. As the fruit matures, starch degrades, soluble sugars such as fructose, glucose, and sucrose increase, cellulose is decomposed, the fruit becomes soft, the tannins are decomposed and the astringency disappears. In stage 3, the content of total sugar and soluble solids reaches the highest, and the fruit is sweeter with the best edibility.

#### 3.3.2 Amino acid content

*A. trifoliata* is a medicinal and edible plant, and different parts have different medical effects (Feng, 2010). Amino acids are essential nutrients for human. *A. trifoliata* fruit contains 17 kinds of amino acids. At four different stages of fruit development, the amino acid content of pulp is shown in **Table 10**. As can be seen from it, during the ripening process of *A. trifoliata* fruit, the amino acid content in the pulp is rich and diverse, and the content of various amino acids generally shows an upward trend. The content of most amino acids reaches the maximum in stage 3, during which a total of 16 free amino acids are detected, including 6 essential amino acids required by human, such as Leu, Ile, etc., as well as 10 of other amino acids, such as Asp and Pro. This result further corroborates the above-mentioned view that stage 3 is the best edible period for *A. trifoliata* fruit. In the optimal stage of fruit taste and nutrition (stage 3), the content of proline acid is the highest, reaching 420mg/100g, followed by aspartic acid (70mg/100g). In addition, the total amino acid content reaches the maximum at stage 3 (860mg/100g), and the best edible period is again proved.

**Table 10:** The content of amino acid in the pulp at different developmental stages of fruit(mg/100g).

Stage	Asp	Thr	Ser	Glu	Pro	Gly	Ala	Cys	Val	Met	Ile	Leu	Tyr	Phe	Lys	His	Arg	Total content
1	50 ± 10c	20 ± 5ab	20 ± 15	60 ± 10	420 ± 20a	20 ± 5	10 ± 0a	20 ± 5a	20 ± 10	0 ± 0a	20 ± 10	40 ± 10	10 ± 0	20 ± 0	30 ± 10	30 ± 0a	10 ± 0b	800
2	40 ± 5bc	10 ± 5b	20 ± 0	70 ± 10	0 ± 0c	20 ± 5	10 ± 0a	20 ± 5a	20 ± 15	0 ± 0a	10 ± 5	30 ± 10	10 ± 5	20 ± 5	20 ± 10	30 ± 5a	10 ± 10b	340
3	70 ± 5ab	30 ± 10a	30 ± 5	50 ± 15	420 ± 40a	30 ± 10	20 ± 10a	20 ± 0a	20 ± 0	0 ± 0a	20 ± 10	40 ± 0	10 ± 5	20 ± 5	30 ± 10	20 ± 5a	30 ± 0a	860
4	60 ± 15a	30 ± 5a	30 ± 10	60 ± 10	260 ± 40b	30 ± 5	20 ± 5a	10 ± 0b	20 ± 10	10 ± 10a	10 ± 5	30 ± 10	10 ± 10	20 ± 5	30 ± 10	20 ± 10a	30 ± 10a	680
*P*	< 0.05	< 0.05	——	——	< 0.05	——	——	< 0.05	——	< 0.05	——	——	——	——	——	——	< 0.05	


Data are presented as mean ± SE (n = 3). Different letters after the values in the same column indicate significant differences among plants the content of amino acid in the pulp at different developmental stages of fruit (P < 0.05).

#### 3.3.3 Organic acid content

Most of the unripe fruits contain a lot of organic acids, so they taste sour. From stage 1 to stage 4, the malic acid content in the pulp is 1.10g/kg, 0.52g/kg, 0.80g/kg and 0.84g/kg, respectively; the lactic acid content in the pulp is 3.30g/kg, 1.00g/kg, 5.00g/kg and 2.30g/kg, respectively; the fumaric acid content in the pulp is 0.0019 g/kg, 0.0000 g/kg, 0.0220 g/kg and 0.0130 g/kg, respectively. As can be seen, *A. trifoliata* generally contains low organic acids, and three organic acids increase first and then decrease, which is basically consistent with the change of organic acids in the post-harvest maturation process of kiwi fruit (Liu et al., 2020).

Malic acid has a soft taste and a special fragrance. It is beneficial to the absorption of amino acids in metabolism, does not accumulate fat, and can present a natural fruit flavor. The acidity of lactic acid is mild and moderate, and has a strong antiseptic and fresh-keeping effect. Fumaric acid has an astringent taste and is one of the solid acids with the strongest acidity. When fumaric acid is changed to sodium fumarate, its water solubility and flavor are better (Wang, 2007). During the ripening process of *A. trifoliata* fruit, the content of various acids is relatively low, and the content of fumaric acid in stage 3 is only 0.0220g/kg. As the fruit matures, some organic acids are converted into sugars, or decomposed by respiratory oxidation, or neutralized into organic salts by potassium and calcium. Therefore, the sourness of the fruit reduces, the sweetness is relatively increased, and the sugar-acid ratio increases as well.

#### 3.3.4 Mineral content

Minerals are important nutrients for human and are of great significance to maintain normal life activities. *A. trifoliata* fruit mainly contains 8 kinds of minerals during the ripening, namely potassium, calcium, phosphorus, magnesium, zinc, iron, copper and manganese. From stage 1 to stage 4, the potassium content in pulp is 1.53g/kg, 1.52g/kg, 1.33g/kg and 2.42g/kg, respectively; the calcium content in pulp is 0.17g/kg, 0.18g/kg, 0.23g/kg and 0.27g/kg, respectively; the content of phosphorus in pulp is 0.21g/kg, 0.22g/kg, 0.21g/kg and 0.32g/kg, respectively; the magnesium content in the pulp is 0.17g/kg, 0.18g/kg, 0.18g/kg and 0.23g/kg, respectively; the content of zinc in pulp is 0.62mg/kg, 0.73mg/kg, 1.29mg/kg and 1.17mg/kg; the content of iron in pulp is 1.29mg/kg, 1.72mg/kg, 4.26mg/kg and 4.49mg/kg, respectively; the copper content in the pulp is 0.89mg/kg, 0.92mg/kg, 0.98mg/kg and 1.37mg/kg, respectively; the content of manganese in pulp is 4.56mg/kg, 5.70mg/kg, 8.39mg/kg and 11.90mg/kg, respectively.

As the fruit matures, the content of calcium, iron, magnesium and manganese shows an upward trend from stage 1 to stage 4. The zinc content reaches a maximum value of 1.29mg/kg at stage 3 as the fruit matures, and then decreases. The potassium content decreases in stage 3, reaching a maximum value of 2.42g/kg in stage 4. The phosphorus content is basically stable from stage 1 to stage 3, but reaches a maximum value of 0.32g/kg in stage 4. In general, during the fruit ripening process, the content of most mineral elements shows the maximum value in the best edible stage 3.

Overall, during the fruit ripening, the content of the main nutrients in the pulp such as total sugar, soluble solids and starch gradually shows an upward trend, and reaches the maximum value in stage 3, which are 19.03g/100g, 22.60%, 5.14g/100g respectively. The total acid content is the least in stage 3, only 0.90g/100g. There are 16 kinds of amino acids in total, including 6 essential amino acids (Lys, Phe, Thr, Ile, Leu and Val), and other 10 kinds of amino acids, such as Asp and Pro. The total amino acid content reaches the maximum value in stage 3, which is 0.86g/100g. The content of organic acid is relatively low, such as 0.0220g/kg fumaric acid. The content of various mineral shows a gradual upward trend during fruit ripening, such as calcium, iron, magnesium and manganese. The results suggest that most of the nutrients in the pulp of stage 3 are the highest, indicating that stage 3 is the best edible period for *A. trifoliata* fruit.

### 3.4 Comprehensive evaluation of fruit quality

The fruit quality indicators were divided into fruit apparent characters and pulp nutrient components to classify and evaluate the fruit quality of the 16 A*. trifoliata* genotypes from Qinba Mountains. The fruit quality parameters include 3 apparent character parameters (single fruit weight, single fruit pulp weight and number of seeds) and 6 nutritional component parameters (total sugar content, vitamin C content, soluble solid content, starch content, total amino acid content, total organic acid content and total mineral content), each of which is divided into 3 grades according to the average value (**Table 11**). Cluster analysis is carried out with SPSS Data Processing System. The grade assignment rules and results are shown in the table below.

**Table 11:** Results of clustering analysis of 16 A*. trifoliata* genotypes with 9 quality indicators (3 grades).

Serial number	Indicator name and unit	Assignment method	A. Trifoliata genotypes	Quantity
1	Total weight of single fruit (g)	grade 1: ≥274.35	HY-9, HY-11	2
		grade 2: 211.40 - 274.34	HY-1, HY-3, HY-4, HY -5, HY-6, HY-7	6
		grade 3: ≤211.39	HY-2, HY-8, HY-8, HY-12, HB-1, HB-2, HB-3, HB-4	8
2	Single fruit pulp weight (g)	grade 1: ≥36.22	HY-2, HY-3	2
		grade 2: 27.64 - 36.21	HY-1, HY-6, HY-7, HY-10, HY-12	5
		grade 3: ≤27.63	HY-4, HY-5, HY-8, HY-9, HY-11, HB-1, HB-2, HB-3, HB-4	9
3	Number of seeds (seed)	grade 1: ≤174	HY-8, HB-1, HB-2, HB-4	4
		grade 2: 175 - 236	HY-2, HY-3, HY- 4, HY-5, HY-6, HY-9, HY-10, HY-12, HB-3	9
		grade 3: ≥237	HY-1, HY-7, HY-11	3
4	Total sugar content (g/100g)	grade 1: ≥16.48	HY-1, HY-2, HY-8, HY-9, HY-12	5
		grade 2: 13.36 - 16.47	HY -4, HY-7, HY-10, HB-1, HB-4	5
		grade 3: ≤13.35	HY-3, HY-5, HY-6, HY-11, HB-2, HB-3	6
5	Vitamin C content (mg/100g)	grade 1: ≥29.76	HY-2, HY-3, HY-5, HY-6, HY-7	5
		grade 2: 19.72 - 29.75	HY-1, HY-4, HY-8, HY-9, HY-10, HY-12, HB-1, HB-3, HB-4	9
		grade 3:.≤19.71	HY-11, HB-2	2
6	Soluble solid content (%)	grade 1: ≥19.03	HY-1, HY-2, HY-8, HY-9, HY-12	5
		grade 2: 15.45 - 19.02	HY-4, HY-5, HY-7, HY-10, HB-1, HB-3, HB-4	7
		grade 3: ≤15.44	HY-3, HY-6, HY-11, HB-2	4
7	Total amino acid content (mg/100g)	grade 1: ≥630.01	HY-1, HY-5, HY-9	3
		grade 2: 420.00 - 630.00	HY-2, HY-8	2
		grade 3: ≤419.99	HY-3, HY-4, HY-6, HY-7, HY-10, HY-11, HY-12, HB-1, HB-2, HB-3, HB-4	11
8	Total organic acid content (g/kg)	grade 1: ≥6.1801	HB-1, HB-2, HB-3, HB-4	4
		grade 2: 3.2301 - 6.1800	HY-1, HY-3, HY-9	3
		grade 3: ≤3.2300	HY-2, HY-4, HY-5, HY-6, HY-7, HY-8, HY-10, HY-11, HY-12	9
9	Total mineral content (mg/kg)	grade 1: ≥2954.56	HY-1, HY-6	2
		grade 2: 2449.44 - 2954.55	HY-2, HY-3, HY-4, HY -8, HY-11, HY-12, HB-1, HB-2, HB-4	9
		grade 3: ≤2449.43	HY-5, HY-7, HY-9, HY-10, HB-3	5

Through the assignment analysis and comprehensive evaluation of 9 quality indicators (3 apparent characters and 6 main chemical components) of the fruits of 16 A*. trifoliata* genotypes from Qinba Mountains, according to the clustering results, the final three excellent genotypes were screened, namely HY-1, HY-2 and HY-9. Among them, HY-1 has three grade 1 parameters, four grade 2 parameters and two grade 3 parameters; HY-2 and HY-9 have four grade 1 parameters, three grade 2 parameters and two grade 3 parameters, respectively.

## 4 Discussion and conclusion

### 4.1 Discussion

Research on *A. trifoliata* mainly focuses on reproduction and cultivation techniques (Bi et al., 2020; Liu et al., 2020). As a new type of fruit, creating excellent varieties that can be used for large-scale promotion and cultivation is the primary problem to be solved. At present, there are not many research on the wild resources and comprehensive and systematic studies on the nutritive components of the wild *A. trifoliata*. In this study, the fruit shape characteristics and pulp nutrient components of 16 A*. trifoliata* genotypes from Qinba Mountains are analyzed and studied. The results show that there are 17 kinds of amino acids in *A. trifoliata* fruit, including 7 essential amino acids and 10 of other amino acids. The organic acids of *A. trifoliata* fruit mainly include malic acid, lactic acid, citric acid and fumaric acid, which give the main flavor to the fruit. Minerals of *A. trifoliata* fruit are mainly calcium, zinc, potassium, iron, copper, phosphorus, magnesium and manganese. These results are basically consistent with the research results of Feng Hang (Feng, 2010), Tang Chenglin et al. (Tang et al., 2021).

The shape characteristics of the fruit and the nutritional components of the pulp are important indicators and basis for evaluating the fruit quality. The data of fruit shape characteristics indicates that *A. trifoliata* germplasm resources in Qinba Mountains show high genetic diversity, which is reflected in the size and shape of the fruit and the proportion of the peel, pulp and seeds. The morphological diversity provides a rich resource for the later development of targeted artificial breeding of *A. trifoliata* (fruit shape, single fruit quality, edible rate, etc.). Through the research on the nutrient components of *A. trifoliata* pulp, it is found that *A. trifoliata* fruit is rich in various nutrients. Based on the medicinal value of *A. trifoliata* itself (Guo et al., 2017; Liu et al., 2020) and the experimental data of fruit nutrients, it shows that *A. trifoliata* has the potential to become a new type of fruit with homology of medicine and food. In the later stage, research on the health-care properties of *A. trifoliata* fruit can be strengthened to explore its value as a new type of health-care fruit. At the same time, research on the breeding of new varieties, storage and preservation and the development of processed products are worthwhile being carried out, which can further provide a theoretical basis for the development of *A. trifoliata* into a new type of characteristic fruit.

### 4.2 Conclusion

1. *A. trifoliata* from Qinba Mountains shows high genetic diversity, which is reflected in the size and shape of the fruit and the proportion of the peel, pulp, and seeds.

2. The maximum single fruit weight of the 16 genotypes is 148.44g (HB-1), and the minimum single fruit weight is 337.29g (HY-11); the maximum longitudinal diameter of the fruit is 156.85mm (HY-9), and the minimum longitudinal diameter is 104.30mm (HY-11); the maximum transverse diameter of the fruit is 62.23mm (HY-1), and the minimum transverse diameter is 49.27mm (HY-8); the maximum thickness of the fruit is 60.26mm (HY-1), and the minimum thickness is 46.78mm (HY-8); the highest edible rate of the fruit is 44.80% (HY-3), and the minimum edible rate is 19.04% (HY-9).

3. The average content of total sugar in the pulp of *A. trifoliata* fruit of the 16 genotypes is 14.68g/100g; the average content of total acid is 0.14g/100g; the average content of vitamin C is 26.40mg/100g; the average content of soluble solids is 17.95%; the average starch content is 5.29g/100g.

4. There are 17 amino acids in *A. trifoliata* fruit, including 7 essential amino acids (Lys, Phe, Met, Thr, Ile, Leu and Val) and 10 other amino acids, 4 kinds of organic acids (the average content of malic acid is 1.03g/kg, lactic acid is 3.38g/kg, citric acid is 0.33g/kg, and fumaric acid is 0.0149g/kg), and 8 kinds of minerals the average content of potassium is 1.83g/kg, calcium is 0.23g/kg, phosphorus is 0.28g/kg, magnesium is 0.21g/kg, zinc is 2.23mg/kg, iron is 2.29mg/kg, copper is 1.37mg/kg, and manganese is 5.52mg/kg.

5. The growth and development of *A. trifoliata*fruit in the later stage can be divided into four stages, and stage 3 is the best edible period. During the ripening process of the fruit, the content of the main nutrients, such as total sugar, gradually shows an upward trend, reaching a maximum value of 19.03g/100g in stage 3; the content of various minerals also shows a gradual upward trend from stage 1 to stage 3, such as calcium, iron, magnesium and manganese. At stage 3, the total sugar and soluble solid reaches the highest, the fruit sweetness increases, and the fruit edible quality is the best.

6. *A. trifoliata* fruits are high in total sugar, vitamin C and soluble solid content, which can improve the flavor. The fruits are also rich in essential amino acids, umami amino acids, sweet amino acids and medicinal amino acids, thus have the potential to become a new type of fruit with homology of medicine and food with the assistance of reasonable ratio. A wide selection of organic acids with less content reduces the sourness of the fruit. Minerals are rich in variety, and the content (such as potassium, zinc and iron) is high and balanced. Therefore, *A. trifoliata* is a new type of economic fruit with high nutritional value and sweet taste, which has broad planting prospects and market potential.

7. Through the comprehensive evaluation of fruit quality of the 16 genotypes of *A. trifoliata* fruit from Qinba Mountains, including 3 parameters of apparent traits and 6 parameters of nutrient components, 3 excellent genotypes are screened out, namely HY-1, HY-2 and HY-9. Then, providing reference for the development and utilization of *A. trifoliata* wild germplasm resources and the selection of new varieties.

## Data availability statement

The original contributions presented in the study are included in the article/**Supplementary Material**. Further inquiries can be directed to the corresponding author.

## Author contributions

MW, X-CG and J-YS conceived and designed the project. MW wrote the manuscript, J-YS revised the manuscript. X-CG and J-YS jointly supervised this work. All authors contributed to the discussion of the results, reviewed the manuscript and approved the final article.

## Funding

This work was supported by the Agricultural Technology Research and Development Project of Xi’an Science and Technology Bureau (Grant No. 21NYYF0012), the Key Research and Development Project of Shaanxi Provincial Department of Science and Technology (Grant No. 2021NY-055), and the Science and Technology Innovation Project of the Academy of Forestry Sciences of Shaanxi Provincial Forestry Department (Grant No. SXLK2021-0211).

## Acknowledgments

We are grateful to the Science and Technology Department of Shaanxi Province for supporting this study. Thanks to Professor Dr. Jean W. H. Yong of the Swedish University of Agricultural Sciences for reviewing the paper and providing constructive comments.

## Conflict of interest

The authors declare that the research was conducted in the absence of any commercial or financial relationships that could be construed as a potential conflict of interest.

## Publisher’s note

All claims expressed in this article are solely those of the authors and do not necessarily represent those of their affiliated organizations, or those of the publisher, the editors and the reviewers. Any product that may be evaluated in this article, or claim that may be made by its manufacturer, is not guaranteed or endorsed by the publisher.
