# Supplementary material for: Analysis of the shape characteristics and nutritional components of Akebia trifoliata in Qinba Mountains
Source: Front Plant Sci. 2022 Sep 29;13:975677. doi: 10.3389/fpls.2022.975677 (PMC9559382; doi:10.3389/fpls.2022.975677)
Supplement: Supplementary file 1 [file DataSheet_1.pdf]

Supplementary Material

1 Supplementary Figures and Tables

1.1 Supplementary Figures

Supplementary Figure 1. Fruit appearance of 16 *Akebia. trifoliata* genotypes

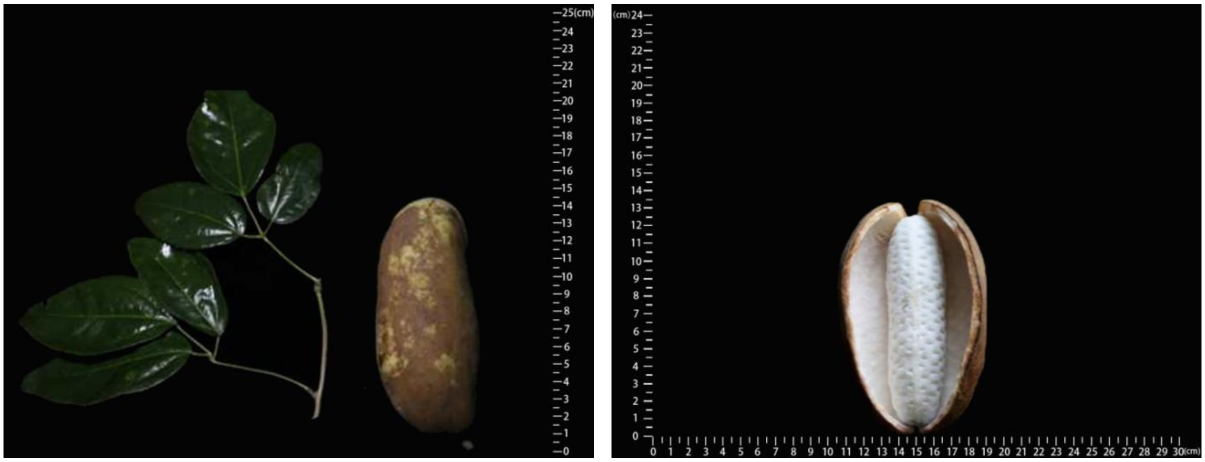

HY-1(Da ye)

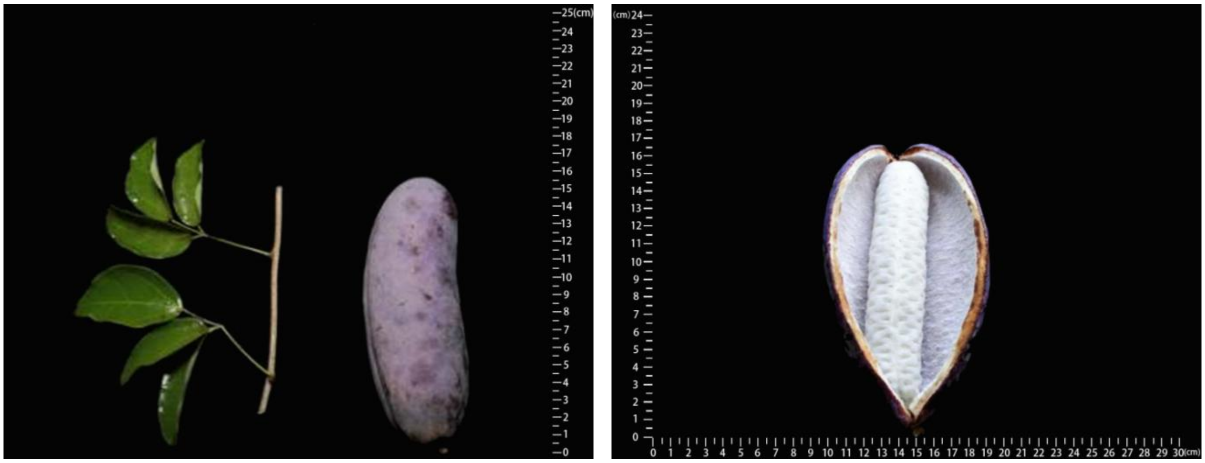

HY-2(Wu yao hua)

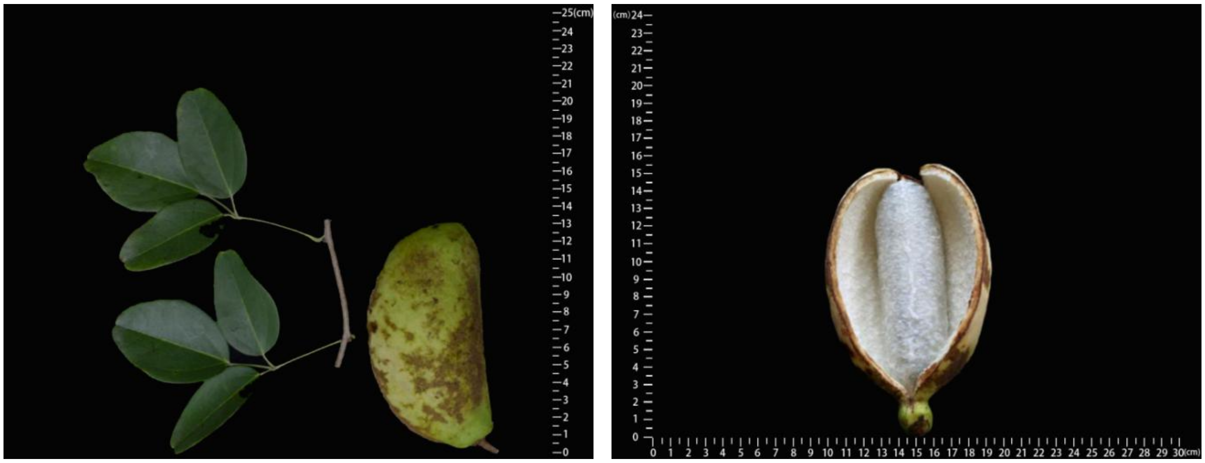

HY-3(Bai huang gua)

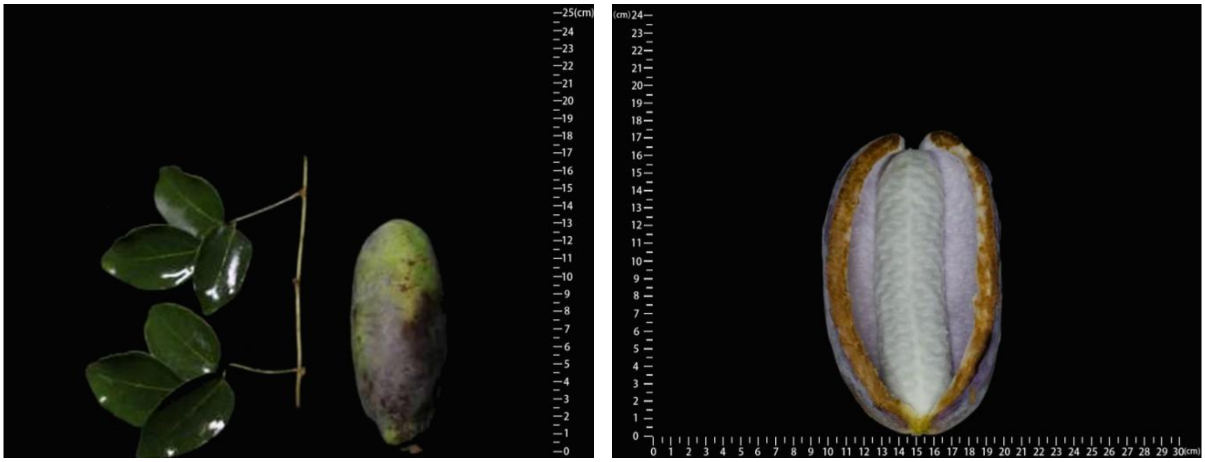

HY-4(Hei zi)

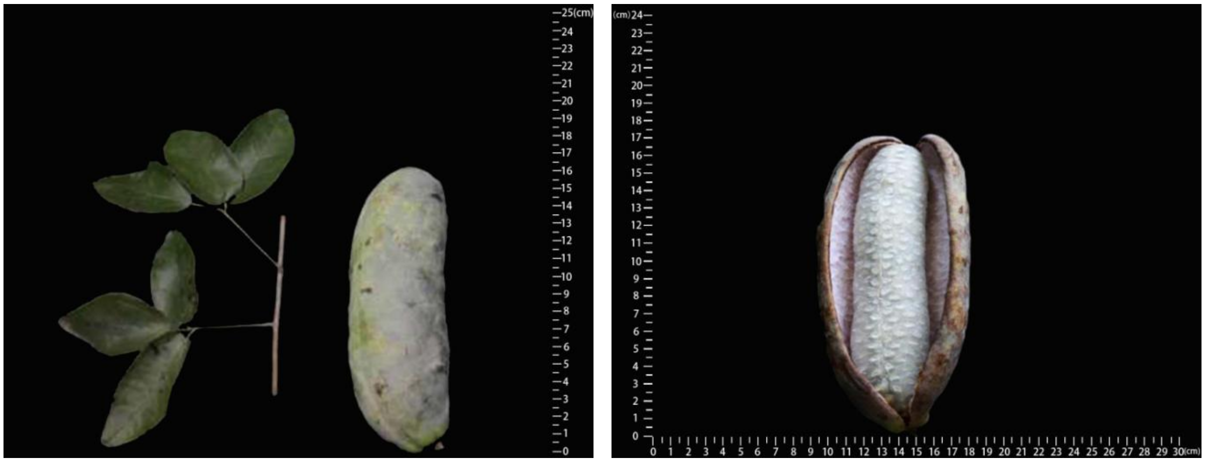

HY-5(Te er)

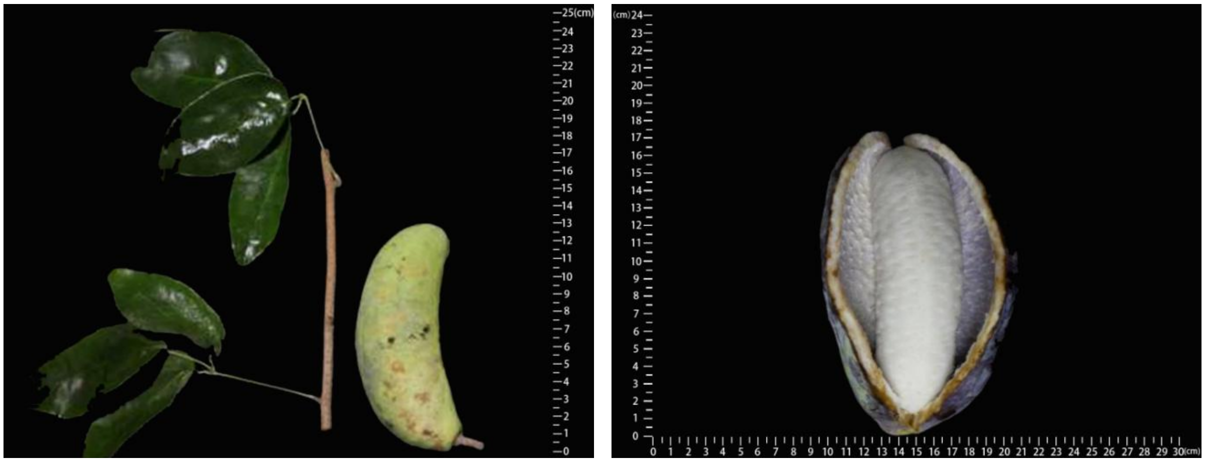

HY-6(Zi ban)

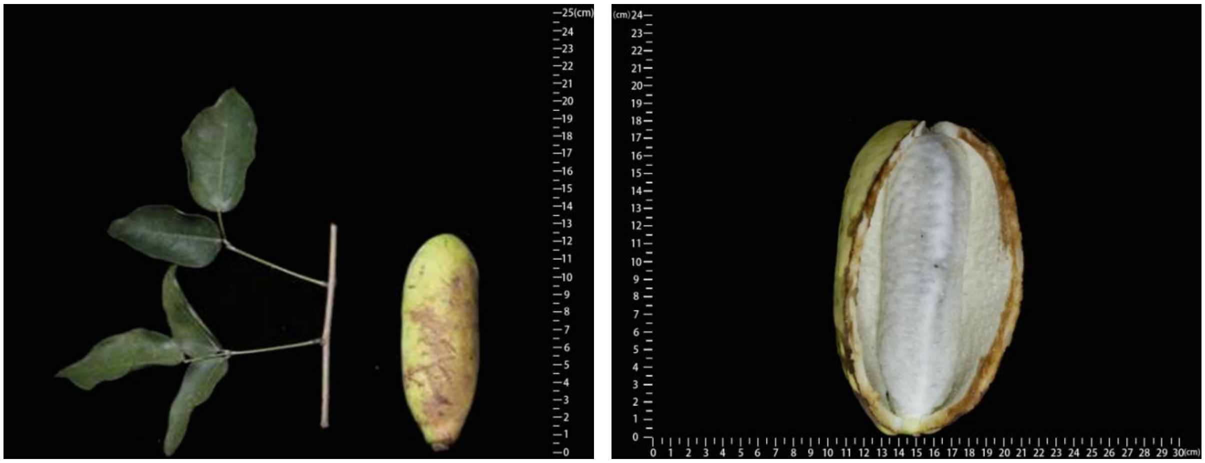

HY-7(Huang bai)

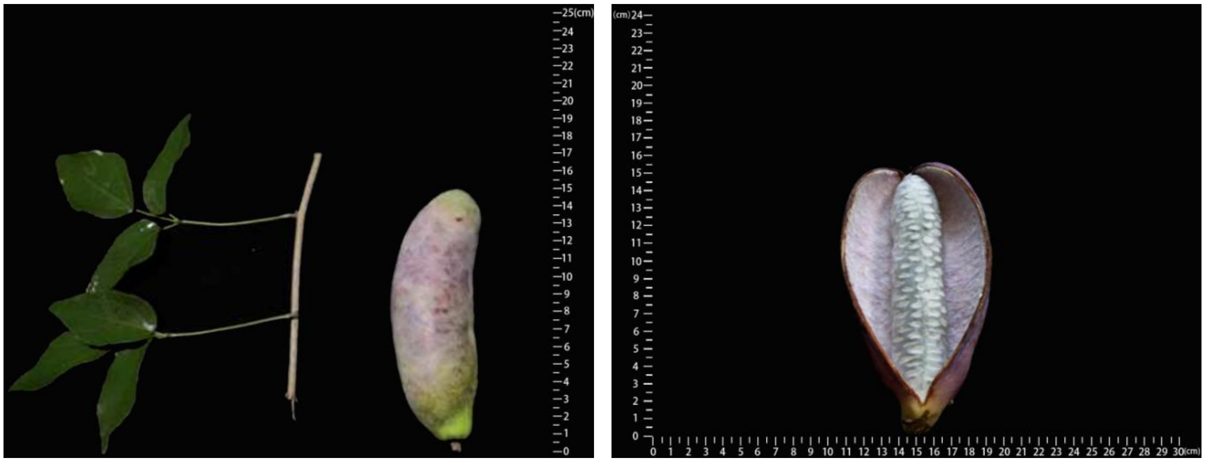

HY-8(Ju hong)

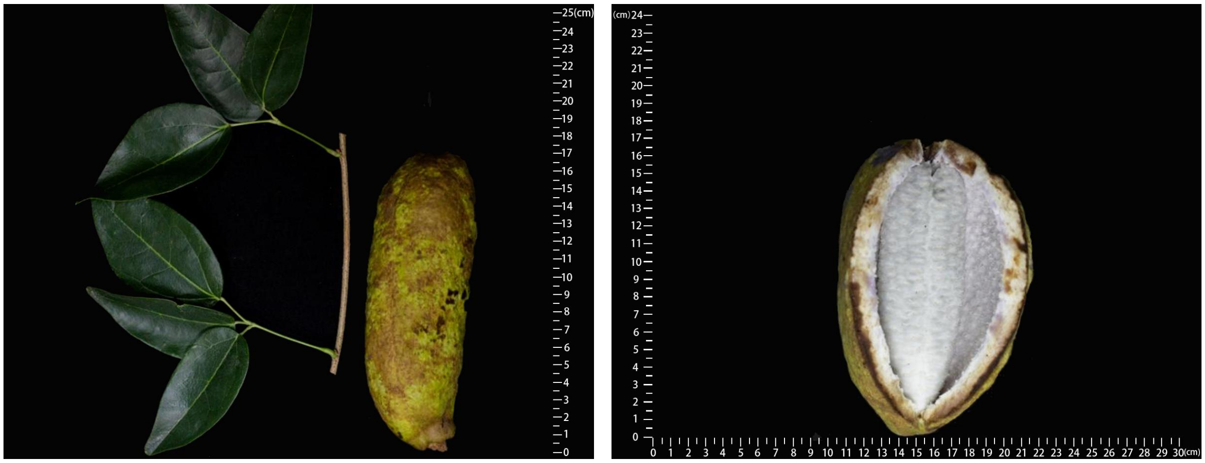

HY-9(Ma pi)

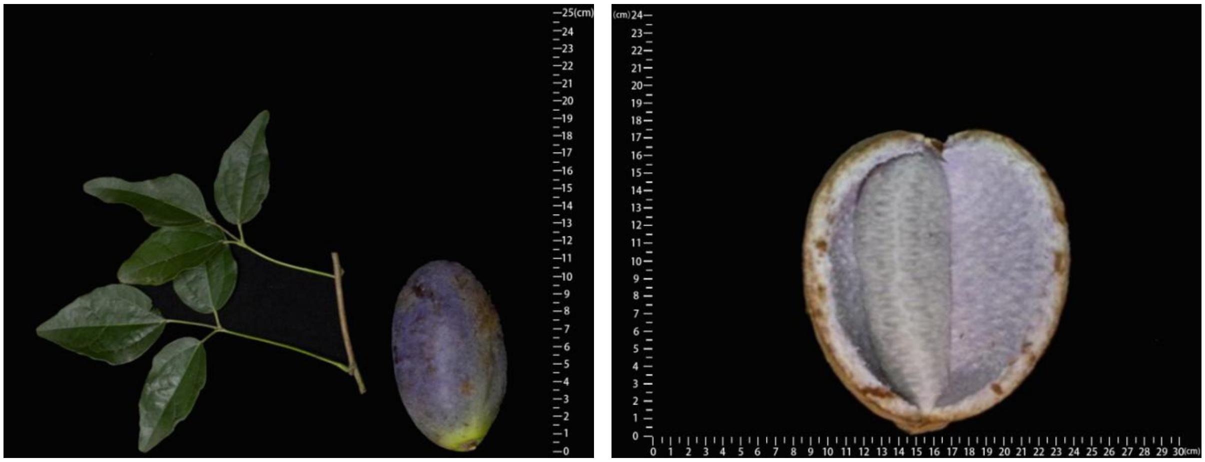

HY-10(Zi bai)

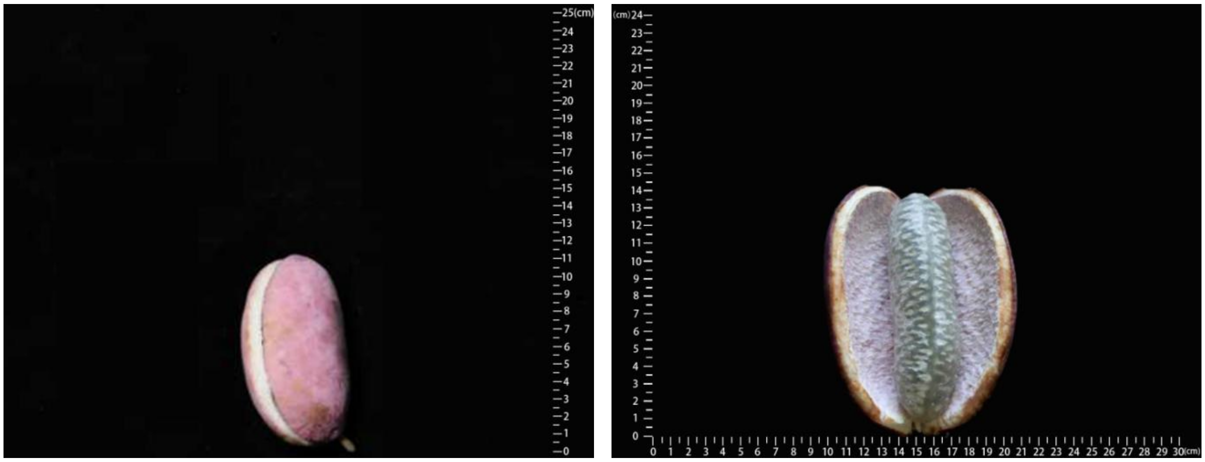

HY-11(Yi hao)

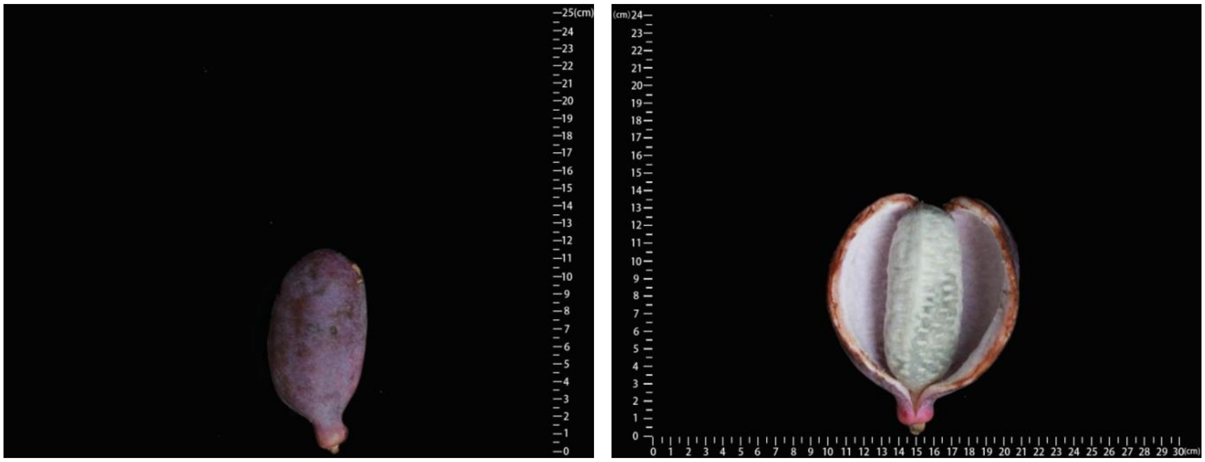

HY-12(Yuan dan dan)

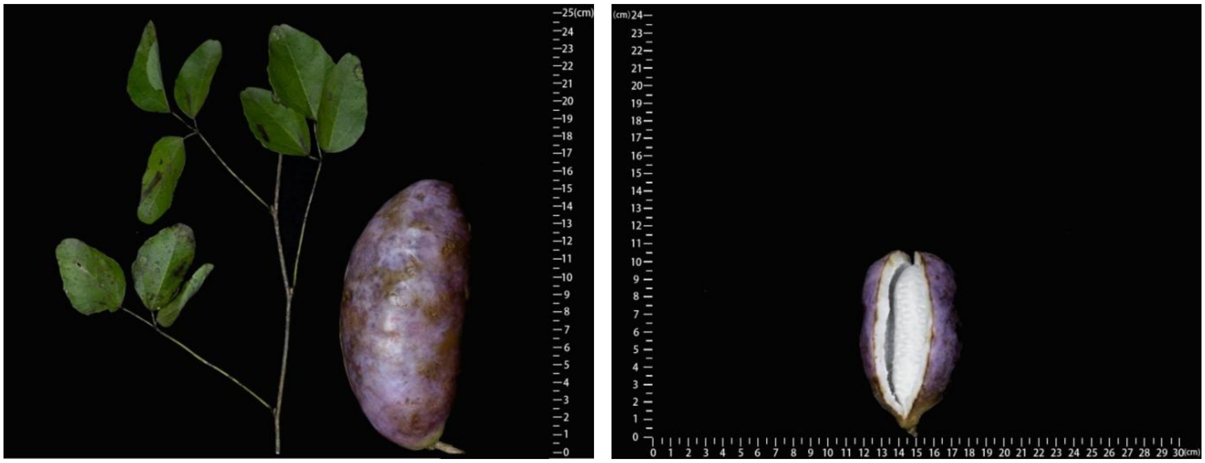

HB-1(Qin zhen yi hao)

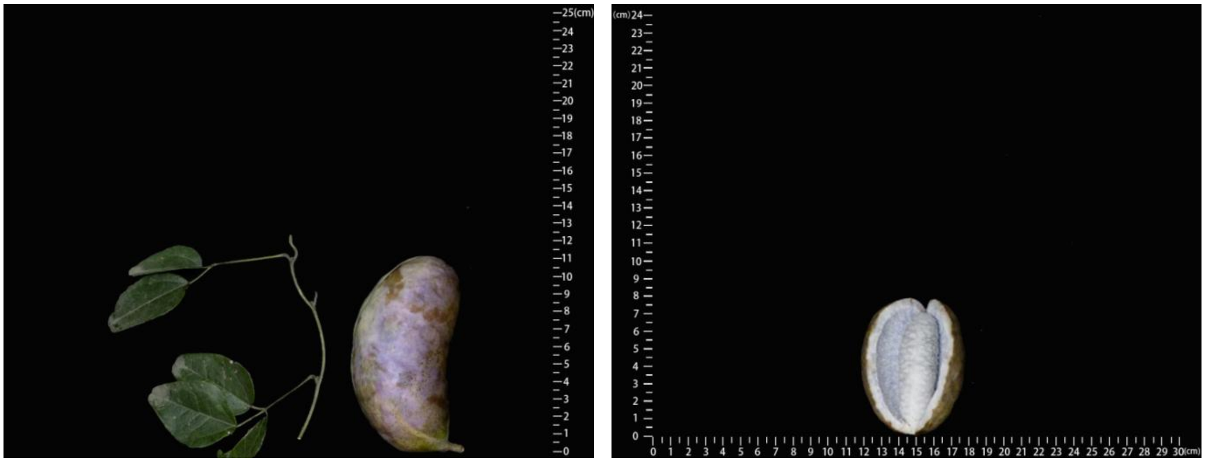

HB-2(Qin zhen er hao)

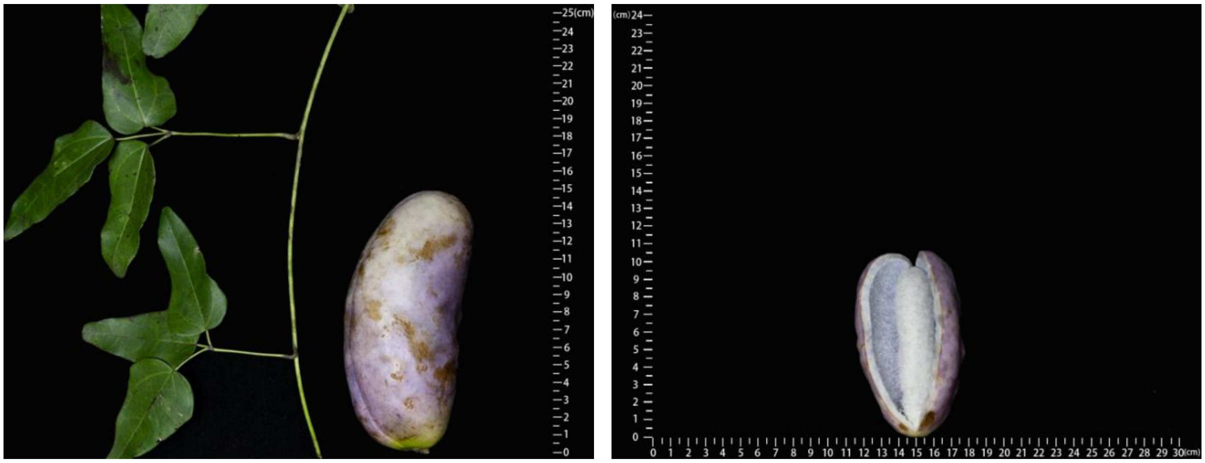

HB-3(Tian zi yi hao)

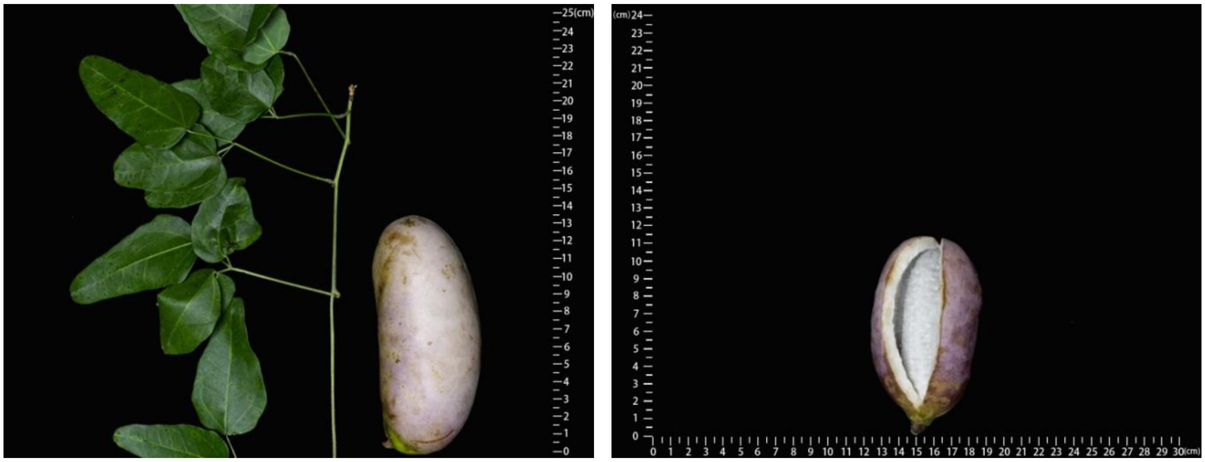

HB-4(Tian zi san hao)

Note: The Chinese names of the genotypes are in brackets.

Supplementary Figure 2. Four stages of *Akebia. trifoliata* fruit development (taking HY-9 material as an example).

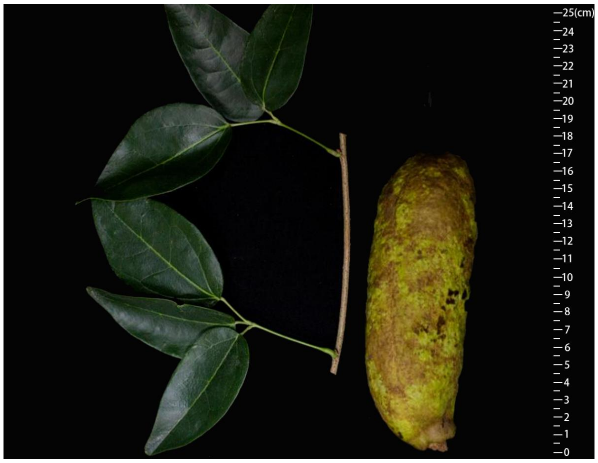

Stage1

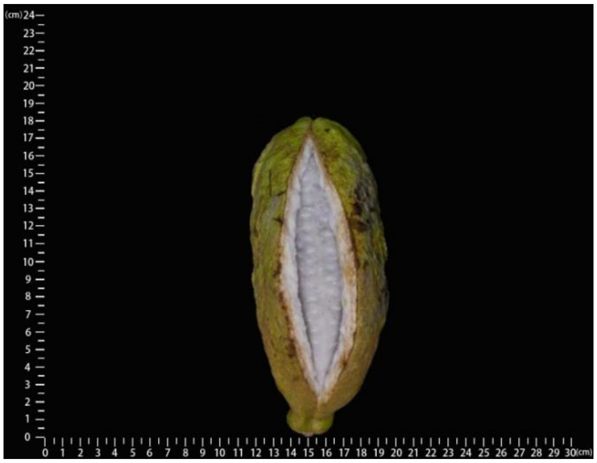

Stage 2

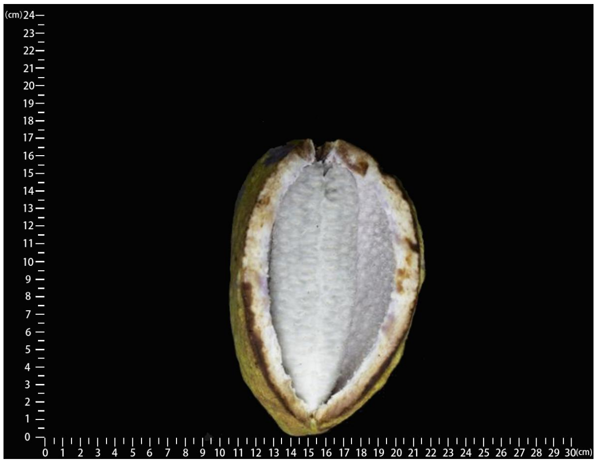

Stage 3

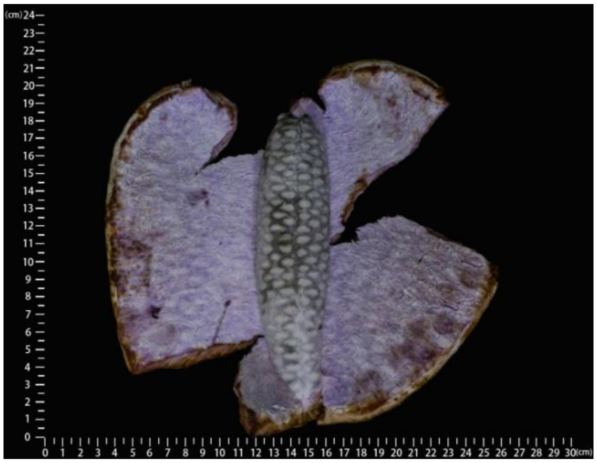

Stage 4

Supplementary Table 1. Results of ANOVA of fruit shape parameters

Name	Source of variation	Sum of square	Degrees of freedom	Mean square	F	Salience
Longitudinal diameter/mm	intergroup	34698.647	10	3469.865	13.374	0.000
	interclass variance	40992.558	158	259.447		
	total	75691.205	168			
Transverse diameter/mm	intergroup	1968.645	10	196.864	9.367	0.000
	interclass variance	3320.726	158	21.017		
	total	5289.370	168			
Thickness/mm	intergroup	2571.977	10	257.198	12.965	0.000
	interclass variance	3134.453	158	19.838		
	total	5706.430	168			

Supplementary Table 2. Results of ANOVA for fruit component parameters

		Sum of square	Degrees of freedom	Mean square	F	Salience
Total weight of single fruit	intergroup	635148.331	15	42343.222	21.197	0.000
	interclass variance	321609.655	161	1997.575		
	total	956757.986	176			
Peel weight of single fruit/g	intergroup	329511.020	15	21967.401	24.988	0.000
	interclass variance	141535.173	161	879.100		
	total	471046.193	176			
Single fruit pulp weight	intergroup	73464.181	15	4897.612	20.980	0.000
	interclass variance	37583.657	161	233.439		
	total	111047.838	176			
Single fruit seed weight/g	intergroup	8886.714	15	592.448	27.850	0.000
	interclass variance	3424.918	161	21.273		
	total	12311.632	176			
Number of seeds per seeds/seed	intergroup	349762.513	15	23317.501	13.818	0.000
	interclass variance	271684.233	161	1687.480		
	total	621446.746	176			
Peel thickness/mm	intergroup	585.338	15	39.023	30.075	0.000
	interclass variance	208.901	161	1.298		
	total	794.239	176			

	intergroup	7814.891	15	520.993	44.777	0.000
Edible rate/%	interclass variance	1873.278	161	11.635		
	total	9688.169	176			

Supplementary Table 3. Results of ANOVA for the content of main chemical components in the pulp of different genotypes of *A. trifoliata* fruit

Name	Source of variation	Sum of square	Degrees of freedom	Mean square	F	Salience
Total sugar (g/100g)	intergroup	339.302	15	22.620	67.018	0.000
	interclass variance	10.801	32	0.338		
	total	350.103	47			
Total acid (g/100g)	intergroup	0.116	15	0.008	744.307	0.000
	interclass variance	0	32	0		
	total	0.117	47			
Vitamin C (mg/100g)	intergroup	2839.861	15	189.324	11943.417	0.000
	interclass variance	0.507	32	0.016		
	total	2840.368	47			
Soluble solids(%)	intergroup	412.928	15	27.529		
	interclass variance	0	32	0		
	total	412.928	47			
Starch(g/100g)	intergroup	236.856	15	15.790	1231.020	0.000
	interclass variance	0.410	32	0.013		
	total	237.266	47			

Supplementary Table 4. Results of ANOVA for the content of essential amino acids in the pulp of different genotypes of *A. trifoliata* fruit

Name	Source of variation	Sum of square	Degrees of freedom	Mean square	F	Salience
Thr	intergroup	2325.000	15	155.000	4.133	0.000
	interclass variance	1200.000	32	37.500		
	total	3525.000	47			
Val	intergroup	525.000	15	35.000	0.622	0.835
	interclass variance	1800.000	32	56.250		
	total	2325.000	47			
Met	intergroup	525.000	15	35.000	11.200	0.000
	interclass variance	100.000	32	3.125		

	total	625.000	47			
Ile	intergroup	1725.000	15	115.000	2.629	0.011
	interclass variance	1400.000	32	43.750		
	total	3125.000	47			
Leu	intergroup	2325.000	15	155.000	3.006	0.004
	interclass variance	1650.000	32	51.563		
	total	3975.000	47			
Phe	intergroup	1200.000	15	80.000	1.707	0.100
	interclass variance	1500.000	32	46.875		
	total	2700.000	47			
Lys	intergroup	1331.250	15	88.750	1.420	0.197
	interclass variance	2000.000	32	62.500		
	total	3331.250	47			

Supplementary Table 5. Results of ANOVA for the content of other amino acids in the pulp of different genotypes of *A. trifoliata* fruit

Name	Source of variation	Sum of square	Degrees of freedom	Mean square	F	Salience
Asp	intergroup	8831.250	15	588.750	7.388	0.000
	interclass variance	2550.000	32	79.688		
	total	11381.25	47			
Ser	intergroup	1725.000	15	115.000	2.538	0.013
	interclass variance	1450.000	32	45.313		
	total	3175.000	47			
Glu	intergroup	6600.000	15	440.000	3.657	0.001
	interclass variance	3850.000	32	120.313		
	total	10450.000	47			
Pro	intergroup	1136925.000	15	75795	527.27	0.000
	interclass variance	4600.000	32	143.75		
	total	1141525.000	47			
Gly	intergroup	600.000	15	40.000	0.692	0.773
	interclass variance	1850.000	32	57.813		
	total	2450.000	47			
Ala	intergroup	525.000	15	35.000	0.933	0.540

	interclass variance	1200.000	32	37.500		
	total	1725.000	47			
	intergroup	1725.000	15	115.000	3.067	0.004
Cys	interclass variance	1200.000	32	37.500		
	total	2925.000	47			
	intergroup	281.25.000	15	18.750	0.632	0.827
Tyr	interclass variance	950.000	32	29.688		
	total	1231.25.000	47			
	intergroup	2831.25.000	15	188.750	2.465	0.016
His	interclass variance	2450.000	32	76.563		
	total	5281.250	47			
	intergroup	17325.000	15	1155.000	19.453	0.000
Arg	interclass variance	1900.000	32	59.375		
	total	19225.000	47			

Supplementary Table 6. Results of ANOVA for the content of organic acid in the pulp of different genotypes of *A. trifoliata* fruit（g/kg）

Name	Source of variation	Sum of square	Degrees of freedom	Mean square	F	Salience
	intergroup	2.848	15	0.190	121.055	0.000
Malic acid	interclass variance	0.050	32	0.002		
	total	2.898	47			
	intergroup	605.118	15	40.341	10579.302	0.000
Lactic acid	interclass variance	0.122	32	0.004		
	total	605.24	47			
	intergroup	6.248	15	0.417	14109.068	0.000
Citric acid	interclass variance	0.001	32	0		
	total	6.249	47			
	intergroup	0.015	15	0.001	2924.443	0.000
Fumaric acid	interclass variance	0	32	0		
	total	0.015	47			

Supplementary Table 7. Results of ANOVA for the content of minerals in the pulp of different genotypes of *A. trifoliata* fruit

Name	Source of variation	Sum of square	Degrees of freedom	Mean square	F	Salience

Potassium (g/kg)	intergroup	5.175	15	0.345	255.138	0.000
	interclass variance	0.043	32	0.001		
	total	5.218	47			
Calcium (g/kg)	intergroup	0.092	15	0.006	61.111	0.000
	interclass variance	0.003	32	0		
	total	0.095	47			
Phosphorus (g/kg)	intergroup	0.223	15	0.015	310.907	0.000
	interclass variance	0.002	32	0		
	total	0.225	47			
Magnesium (g/kg)	intergroup	0.163	15	0.011	520.72	0.000
	interclass variance	0.001	32	0		
	total	0.163	47			
Zinc (mg/kg)	intergroup	1098.285	15	73.219	0.933	0.540
	interclass variance	2511.729	32	78.492		
	total	3610.014	47			
Iron (mg/kg)	intergroup	40.264	15	2.684	908.642	0.000
	interclass variance	0.095	32	0.003		
	total	40.359	47			
Copper (mg/kg)	intergroup	10.450	15	0.697	723.837	0.000
	interclass variance	0.031	32	0.001		
	total	10.481	47			
Manganese (mg/kg)	intergroup	159.677	15	10.645	541.794	0.000
	interclass variance	0.629	32	0.020		
	total	160.305	47			

Supplementary Table 8. Results of ANOVA for the content of main nutrients in the pulp at different developmental stages of fruit

Name	Source of variation	Sum of square	Degrees of freedom	Mean square	F	Salience
Total sugar	intergroup	67.195	3	22.398	18553.282	0.000
	interclass variance	0.010	8	0.001		
	total	67.205	11			
Acid value	intergroup	0.142	3	0.047	8536.017	0.000
	interclass variance	0	8	0		

	total	0.142	11			
Vitamin C	intergroup	72.843	3	24.281	18210.706	0.000
	interclass variance	0.011	8	0.001		
	total	72.853	11			
Soluble solids	intergroup	76.222	3	25.407	84691.667	0.000
	interclass variance	0.002	8	0		
	total	76.225	11			
Starch	intergroup	9.305	3	3.102	2406.957	0.000
	interclass variance	0.010	8	0.001		
	total	9.315	11			

Supplementary Table 9. Results of ANOVA for the content of amino acid in the pulp at different developmental stages of fruit(mg/100g)

Name	Source of variation	Sum of square	Degrees of freedom	Mean square	F	Salience
Asp	intergroup	1500.000	3	500.000	5.333	0.026
	interclass variance	750.000	8	93.750		
	total	2250.000	11			
Thr	intergroup	825.000	3	275.000	6.286	0.017
	interclass variance	350.000	8	43.750		
	total	1175.000	11			
Ser	intergroup	300.000	3	100.000	1.143	0.389
	interclass variance	700.000	8	87.500		
	total	1000.000	11			
Glu	intergroup	600.000	3	200.000	1.524	0.281
	interclass variance	1050.000	8	131.250		
	total	1650.000	11			
Pro	intergroup	353700.000	3	117900.000	131.000	0.000
	interclass variance	7200.000	8	900.000		
	total	360900.000	11			
Gly	intergroup	300.000	3	100.000	2.286	0.156
	interclass variance	350.000	8	43.750		
	total	650.000	11			
Ala	intergroup	300.000	3	100.000	3.200	0.084
	interclass variance	250.000	8	31.250		

	total	550.000	11			
	intergroup	225.000	3	75.000	6.000	0.019
Cys	interclass variance	100.000	8	12.500		
	total	325.000	11			
	intergroup	0.000	3	0.000	0.000	1.000
Val	interclass variance	850.000	8	106.250		
	total	850.000	11			
	intergroup	225.000	3	75.000	3.000	0.095
Met	interclass variance	200.000	8	25.000		
	total	425.000	11			
	intergroup	300.000	3	100.000	1.600	0.264
Ile	interclass variance	500.000	8	62.500		
	total	800.000	11			
	intergroup	300.000	3	100.000	1.333	0.330
Leu	interclass variance	600.000	8	75.000		
	total	900.000	11			
	intergroup	0.000	3	0.000	0.000	1.000
Tyr	interclass variance	300.000	8	37.500		
	total	300.000	11			
	intergroup	0.000	3	0.000	0.000	1.000
Phe	interclass variance	150.000	8	18.750		
	total	150.000	11			
	intergroup	300.000	3	100.000	1.000	0.441
Lys	interclass variance	800.000	8	100.000		
	total	1100.000	11			
	intergroup	300.000	3	100.000	2.667	0.119
His	interclass variance	300.000	8	37.500		
	total	600.000	11			
	intergroup	1200.000	3	400.000	8.000	0.009
Arg	interclass variance	400.000	8	50.000		
	total	1600.000	11			

Supplementary Table 10. Organic acid content of pulp at different developmental stages of fruit(g/kg)

Stage	Malic acid	Lactic acid	Fumaric acid
1	1.10±0.04a	3.30±0.02b	0.0019±0.0001c
2	0.52±0.01c	1.00±0.02d	0.0000±0.0000d
3	0.80±0.04b	5.00±0.06a	0.022±0.0009a
4	0.84±0.02b	2.3±0.03c	0.013±0.0003b

Data are presented as mean ± SE (n = 3). Different letters after the values in the same column indicate significant differences among plants the organic acid content of pulp at different developmental stages of fruit ($P < 0.05$)

Supplementary Table 11. Results of ANOVA for the organic acid content of pulp at different developmental stages of fruit(g/kg)

Name	Source of variation	Sum of square	Degrees of freedom	Mean square	F	Salience
Malic acid	intergroup	0.531	3	0.177	184.523	0
	interclass variance	0.008	8	0.001		
	total	0.538	11			
Lactic acid	intergroup	25.467	3	8.489	2741.242	0
	interclass variance	0.025	8	0.003		
	total	25.492	11			
Fumaric acid	intergroup	0.001	3	0.000	1280.024	0
	interclass variance	0.000	8	0.000		
	total	0.001	11			

Supplementary Table 12. Mineral content of pulp at different developmental stages of fruit

Stage	Potassium (g/kg)	Calcium(g/kg)	Phosphorus(g/kg)	Magnesium(g/kg)	Zinc(mg/kg)	Iron(mg/kg)	Copper(mg/kg)	Manganese(mg/kg)
1	1.53±0.01b	0.17±0.00c	0.21±0.01c	0.17±0.00c	0.62±0.01d	1.29±0.00d	0.89±0.01d	4.56±0.02d
2	1.52±0.05b	0.18±0.01c	0.22±0.00b	0.18±0.01b	0.73±0.01c	1.72±0.09c	0.92±0.02c	5.70±0.13c
3	1.33±0.00c	0.23±0.01b	0.21±0.01c	0.18±0.00b	1.29±0.01a	4.26±0.01b	0.98±0.01b	8.39±0.03b
4	2.42±0.02a	0.27±0.01a	0.32±0.01a	0.23±0.00a	1.17±0.04b	4.49±0.13a	1.37±0.01a	11.9±0.13a

Data are presented as mean ± SE (n = 3). Different letters after the values in the same column indicate significant differences among plants the mineral content of pulp at different developmental stages of fruit ($P < 0.05$)

Supplementary Table 13. Results of ANOVA for the mineral content of pulp at different developmental stages of fruit

Name	Source of variation	Sum of square	Degrees of freedom	Mean square	F	Salience
Potassium (g/kg)	intergroup	2.135	3	0.712	1004.906	0.000
	interclass variance	0.006	8	0.001		

	total	2.141	11			
	intergroup	0.019	3	0.006	248.000	0.000
Calcium(g/kg)	interclass variance	0.017	1	0.017	693.600	0.000
	total	0.019	11			
	intergroup	0.027	3	0.009	216.267	0.000
Phosphorus(g/kg)	interclass variance	0.000	8	0.000		
	total	0.027	11			
	intergroup	0.006	3	0.002	257.000	0.000
Magnesium(g/kg)	interclass variance	0.000	8	0.000		
	total	0.006	11			
	intergroup	0.940	3	0.313	854.303	0.000
Zinc(mg/kg)	interclass variance	0.003	8	0.000		
	total	0.943	11			
Iron(mg/kg)	intergroup	25.118	3	8.373	1385.835	0.000
	interclass variance	0.048	8	0.006		
	total	25.167	11			
	intergroup	0.449	3	0.150	946.158	0.000
Copper(mg/kg)	interclass variance	0.001	8	0.000		
	total	0.451	11			
	intergroup	96.469	3	32.156	4582.864	0.000
Manganese(mg/kg)	interclass variance	0.056	8	0.007		
	total	96.525	11			

Supplementary Table 14. Evaluation index of *Akebia. trifoliata* fruit quality

Serial number	Material	The total weight of a single fruit (g)	Single fruit pulp weight (g)	Number of seeds (seeds)	Total sugar content (100g)	Vitamin C content (mg/100g)	Soluble solid content (%)	Total amino acid content (mg/100g)	Total organic acid content (g/kg)	Total mineral content (mg/kg)
1	HY-1	273.37	34.62	247	16.67	24.21	19.08	800.00	4.23	3459.66
2	HY-2	208.15	44.27	179	17.07	36.25	20.39	580.00	2.43	2786.71
3	HY-3	213.19	44.80	206	10.79	30.29	11.86	400.00	5.42	2493.21
4	HY-4	263.95	25.63	224	15.70	29.66	18.60	340.00	2.08	2593.90
5	HY-5	241.7	25.42	212	13.15	33.80	18.10	700.00	2.76	2424.47
6	HY-6	245.02	29.87	179	10.22	35.91	14.70	350.00	3.18	3093.76

7	HY-7	221.21	36.11	330	14.36	39.80	18.50	370.00	1.80	1944.32
8	HY-8	183.73	25.45	143	17.55	27.90	22.00	590.00	1.76	2938.10
9	HY-9	327.47	19.04	221	19.03	23.76	22.60	860.00	5.82	1960.92
10	HY-10	175.13	34.08	190	13.72	29.63	18.00	310.00	2.10	2146.38
11	HY-11	337.29	27.37	287	12.84	9.66	13.56	180.00	2.04	2486.64
12	HY-12	183.98	32.17	193	19.60	22.31	21.57	360.00	1.77	2901.07
13	HB-1	148.44	23.39	157	14.48	20.26	16.10	340.00	10.30	2478.99
14	HB-2	149.39	24.61	160	13.26	18.29	15.20	300.00	10.60	2598.73
15	HB-3	188.73	26.62	180	12.40	20.93	18.20	370.00	9.72	2080.90
16	HB-4	153.66	22.30	149	14.05	19.72	18.70	330.00	10.05	2711.38

Supplementary Table 15. Normal distribution test of evaluation index of *Akebia. trifoliata* fruit quality

Hypothesis Testing Summary

	Null hypothesis	Test	Salience	Decision making
1	The distribution of the total weight of a single fruit is normally distributed with a mean of 219.65 and a standard deviation of 58.58418.	One-Sample Kolmogorov-Sminov Test	0.200[a,b]	Keep the null hypothesis
2	The distribution of single fruit pulp weight is normally distributed with a mean of 29.73 and a standard deviation of 7.45147.	One-Sample Kolmogorov-Sminov Test	0.138[a]	Keep the null hypothesis
3	The distribution of number of seeds is normal distribution with a mean of 204 and a standard deviation of 50.598.	One-Sample Kolmogorov-Sminov Test	0.200[a,b]	Keep the null hypothesis
4	The distribution of total sugar content is normally distributed with a mean of 14.68 and a standard deviation of 2.72647.	One-Sample Kolmogorov-Sminov Test	0.200[a,b]	Keep the null hypothesis
5	The distribution of vitamin C content is normally distributed with a mean of 26.40 and a standard deviation of 7.94549.	One-Sample Kolmogorov-Sminov Test	0.200[a,b]	Keep the null hypothesis
6	The distribution of soluble solid content is normally distributed with a mean of 17.95 and a standard deviation of 3.02922.	One-Sample Kolmogorov-Sminov Test	0.108[a]	Keep the null hypothesis
7	The distribution of total amino acid content is normally distributed with a mean of 449 and a standard deviation of 196.057.	One-Sample Kolmogorov-Sminov Test	0.001[a]	Keep the null hypothesis
8	The distribution of total organic acid content is normally distributed with a mean of 4.75 and a standard deviation of 3.45934.	One-Sample Kolmogorov-Sminov Test	0.016[a]	Keep the null hypothesis
9	The distribution of total mineral content is normally distributed with a mean of 2568.70 and a standard deviation of 416.54767.	One-Sample Kolmogorov-Sminov Test	0.200[a,b]	Keep the null hypothesis

Asymptotic significance is shown. The significance level is 0.050.
a. After Lilley correction.
b. This is the lower bound for true significance.

Supplementary Table 16. Results of clustering analysis of 16 *A. trifoliata* genotypes with 9 quality indicators (3 grades)

Serial number	Material	Total weight of single fruit	Single fruit pulp weight	Number of seeds	total sugar content	Vitamin C content	Soluble solids content	Total amino acid content	Total organic acid content	Total mineral content
1	HY-1	2	2	3	1	2	1	1	2	1
2	HY-2	3	1	2	1	1	1	2	3	2
3	HY-3	2	1	2	3	1	3	3	2	2
4	HY-4	2	3	2	2	2	2	3	3	2
5	HY-5	2	3	2	3	1	2	1	3	3
6	HY-6	2	2	2	3	1	3	3	3	1
7	HY-7	2	2	3	2	1	2	3	3	3
8	HY-8	3	3	1	1	2	1	2	3	2
9	HY-9	1	3	2	1	2	1	1	2	3
10	HY-10	3	2	2	2	2	2	3	3	3
11	HY-11	1	3	3	3	3	3	3	3	2
12	HY-12	3	2	2	1	2	1	3	3	2
13	HB-1	3	3	1	2	2	2	3	1	2
14	HB-2	3	3	1	3	3	3	3	1	2
15	HB-3	3	3	2	3	2	2	3	1	3
16	HB-4	3	3	1	2	2	2	3	1	2

Supplementary Table 17. The grades chart of 9 fruit quality parameters

Total weight of single fruit

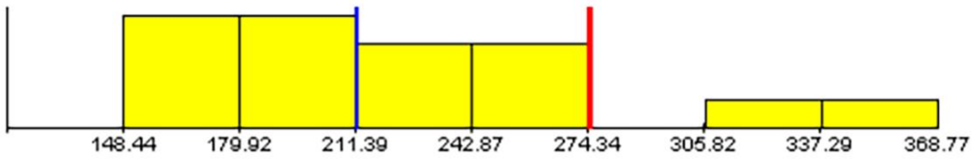

Single fruit pulp weight

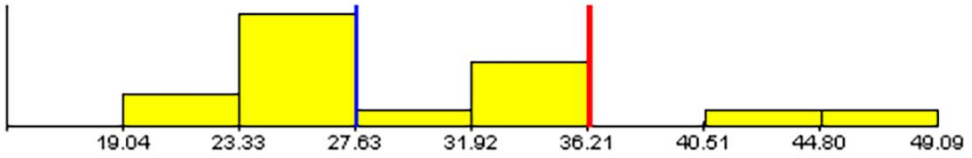

Number of seeds

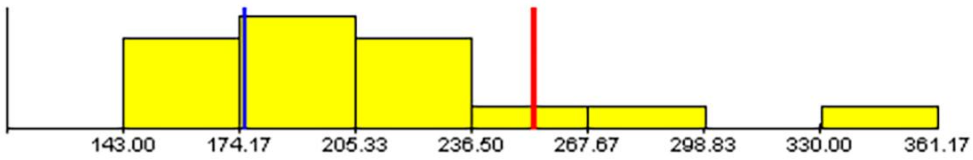

total sugar content

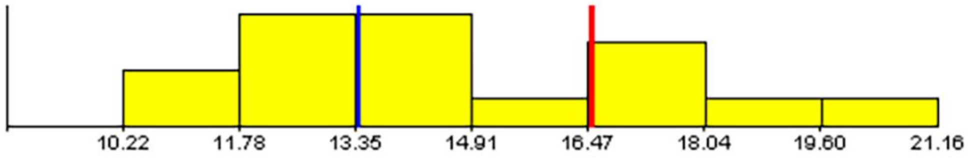

Vitamin C content

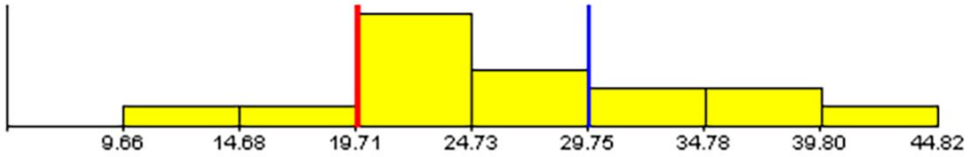

Soluble solids content

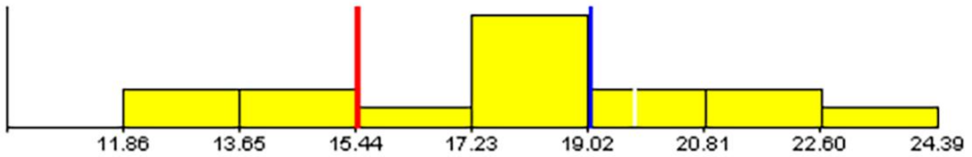

Total amino acid content

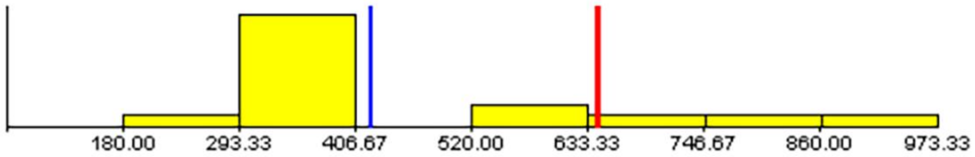

Total organic acid content

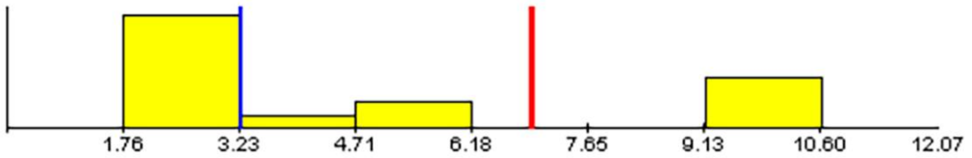

Total mineral content

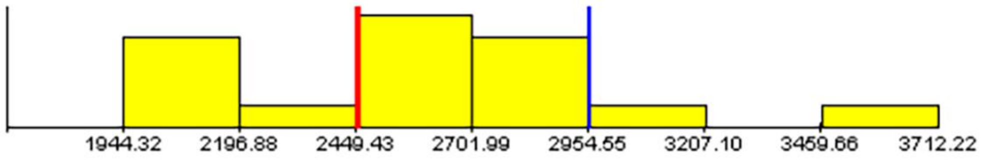
1944.32
2196.88
2449.43
2701.99
2954.55
3207.10
3459.66
3712.22
